# The control of movement gradually transitions from feedback control to feedforward adaptation throughout childhood

**DOI:** 10.1038/s41539-025-00304-7

**Published:** 2025-03-11

**Authors:** Laura A. Malone, Nayo M. Hill, Haley Tripp, Vadim Zipunnikov, Daniel M. Wolpert, Amy J. Bastian

**Affiliations:** 1 Kennedy Krieger Institute, Baltimore, MD USA; 2 Department of Neurology, Johns Hopkins School of Medicine, Baltimore, MD USA; 3 Department of Physical Medicine and Rehabilitation, Johns Hopkins School of Medicine, Baltimore, MD USA; 4 Department of Neuroscience, Johns Hopkins School of Medicine, Baltimore, MD USA; 5 Department of Biostatistics, Johns Hopkins Bloomberg School of Public Health, Baltimore, MD USA; 6 Mortimer B. Zuckerman Mind Brain Behavior Institute, Columbia University, New York, NY USA; 7 Department of Neuroscience, Columbia University, New York, NY USA

**Keywords:** Learning and memory, Motor control

## Abstract

The ability to adjust movements in response to perturbations is key for an efficient and mature nervous system, which relies on two complementary mechanisms — feedforward adaptation and feedback control. We examined the developmental trajectory of how children employ these two mechanisms using a previously validated visuomotor rotation task, conducted remotely in a large cross-sectional cohort of children aged 3–17 years and adults (*n* = 656; 353 males & 303 females). Results revealed a protracted developmental trajectory, with children up to ~13–14 years showing immature adaptation. Younger children relied more on feedback control to succeed. When adaptation was the only option, they struggled to succeed, highlighting a limited ability to adapt. Our results show a gradual shift from feedback control to adaptation learning throughout childhood. We also generated percentile curves for adaptation and overall performance, providing a reference for understanding the development of motor adaptation and its trade-off with feedback control.

## Introduction

Adults are adept at counteracting perturbations to their motor system to achieve their goals, but this is not an innate ability. Infants as young as 3- to 6-months old can reach to toys using jerky, swatting movements^1,2^; yet, is it only after 2–3 years of moving in this way that a child’s reaching pattern begins to become more smooth and stereotyped^3^. It is not until adulthood when individuals exhibit exquisite flexibility in their reaching movements even when faced with unexpected perturbations and different environments^4^.

The ability to respond to errors that arise during movements relies on two complementary mechanisms — feedforward adaptation and feedback control. Consider a child playing soccer in wind that blows consistently across the field. When the child tries to pass the ball to another player, if they aim directly at their team-mate, the ball will deviate due to the wind and not arrive at its desired destination. Once the ball is kicked, the player has no more control over its path. However, errors can be used to drive adaptation and improve subsequent passes by taking into account the effect of the wind. That is the player should learn to deviate their kick direction into the wind so that the pass reaches its desired location. Such feedforward adaptation occurs on a time scale of minutes to hours, and is thought to update an internal model representing the perturbation^5,6^. A hallmark of such learning is that when the perturbation is removed (a sudden lull), there will be an aftereffect in that the ball will miss its intended target in the opposite direction to the initial errors^7^.

In contrast to passing the ball, when dribbling, there is an additional mechanism that can be used for control. As in passing, each touch of the ball could be adapted so as to deviate the kick into the wind. However, dribbling also allows feedback control to be used. If there is no adaptation to the wind, the ball may drift off the desired path and visual feedback of the ensuing error can be used to compensate and return to the intended direction. Therefore, dribbling in the face of the wind could be achieved by different combinations of feedforward adaptation and feedback control.

Adaptation is thought to rely on the cerebellum. For example, people with damage to the cerebellum^8–12^, make uncoordinated and inaccurate movements (i.e., ataxia) and have deficits in adaptation. The current thinking is that the cerebellum is involved in housing internal models of sensorimotor control and in recalibrating them via adaptation^13^. When this process is disrupted, people with cerebellar damage have to rely more on feedback-based corrections during movement^14,15^. A benefit of this is that adjustments can occur within a movement. However, these movement corrections rely on time-delayed sensory signals (e.g., proprioceptive long-latency response delays are 50–105 ms, visual delay is 150–180 ms)^15–17^. These delays impact the speed, accuracy, and automatization of movement.

There is evidence that the ability to use adaptation changes through childhood^18^, possibly due in part to protracted maturation of the cerebellum. Prior work in walking suggests that adaptation may not be adult-like until adolescence^18^. Some studies suggest reaching adaptation might be mature at an earlier age (i.e., in school age children)^19,20^. Other studies have suggested that younger children (~5–6 years of age) require greater cognitive attention on their arm movements, plan shorter movement segments, and have more variable movements^21–23^, which suggests that they rely more on feedback control.

Here we examine the development across childhood of adaptation and feedback control in two tasks which are analogous to dribbling (guiding a ball; Push ball) and passing (throwing a ball; Launch ball). Across trials, a visuomotor rotation was gradually introduced between the motion of the hand and the ball. Push ball allows us to quantify both adaptation of the initial reach direction as well as feedback corrections later in the reach. In this task, each participant can vary in their use of adaptation versus feedback mechanisms. Launch ball allows us to isolate adaptation because feedback control is not possible. We can then assess whether children who rely on feedback control in Push ball do so out of choice (i.e., it is easier) or necessity (i.e., they cannot use adaptation). Finally, from our large sample of over 600 participants, we develop growth curves for features of sensorimotor control across childhood. This was done to allow researchers insight into the development of adaptation throughout childhood and the tradeoff with feedback control.

## Results

We created an online video game to investigate the development of adaptation versus use of online feedback control across childhood. The game used a colorful, entertaining, 3D environment to make the experiment fun and engaging for children from toddler-age through adolescence (previously described and validated in Malone et al.^24^). At the start of each trial, a still image of a different Disney cartoon video clip was presented on the screen above the target (a play button). Participants used an input device (mouse, trackpad, touchscreen; we use mouse as the generic term for these) with their dominant hand and moved the ball on a computer screen with the goal of contacting the target. If the participant hit the target the short cartoon clip would play; if they missed, a sad face and the word “Miss” appeared, the ball turned red, and the video did not play.

Children aged 3–17 years and adults (cross sectional sample, *n* = 656) participated by playing one of two versions of the game. In *Push ball*, the motion of the ball was guided by the motion of the hand throughout the trial. In contrast, in *Launch ball*, the motion of the ball was launched along a straight path based on the participant’s hand movement direction. Once launched, the ball continued in its launched direction and could not be altered by motion of the participant’s hand. In the absence of a perturbation, the ball followed the mouse movement in a similar way to controlling the cursor on a computer (Push ball) or was launched in the direction of normal cursor movement (Launch ball). In contrast, when we introduced a perturbation in the form of a visuomotor rotation, the ball’s motion or launch direction was rotated from the mouse movement.

**Fig. 1:**
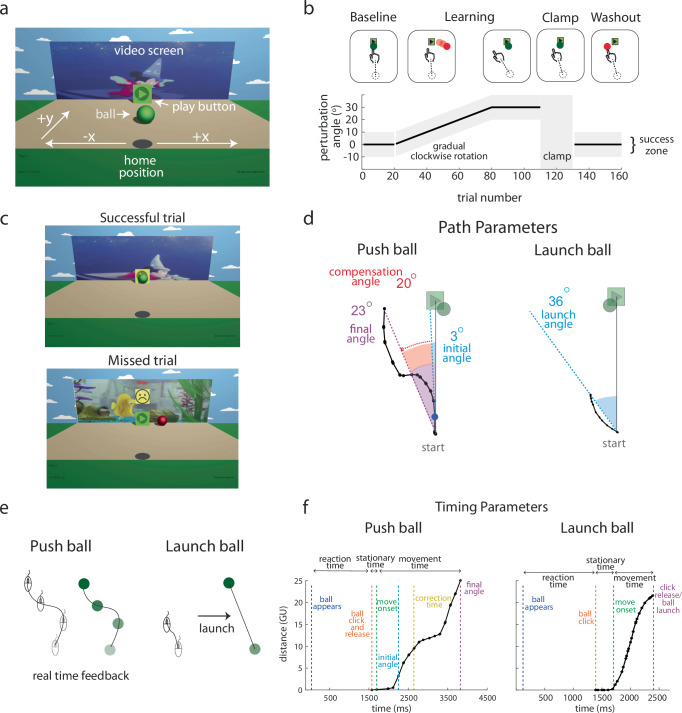
Experimental setup and paradigm for Push ball and Launch ball. Experimental setup and paradigm. **a** The game environment was displayed on a computer screen, with white text and arrows hidden from the participants. **b** The task consisted of 160 trials (Push ball) or 180 trials (Launch ball) across four blocks: baseline, learning, error clamp, and washout. The plot shows the time course of the visuomotor rotation. Gray zones indicate the range of reach angles that would successfully hit the play button. Brief rest breaks occurred between the baseline and learning blocks and between the error clamp and washout blocks. **c** Visual scenes shown to participants for successful (top) and unsuccessful (missed; bottom) trials. **d** Key performance parameters for Push ball and Launch ball. The hand path (black line) from a trial late in the learning block (30-degree perturbation) is shown. In Push ball, the *final angle* (purple) is the angle between the start-to-target line and the line connecting the start and end of the hand path. The *initial angle* (blue) is the angle from start to a point 5% of the distance along the path, reflecting feedforward control. The *compensation angle* (red) is the difference between final and initial angles, representing online visual feedback use. In Launch ball, the launch angle (blue) is the angle between the start-to-target line and the hand path’s start-to-end line. **e** A schematic illustrates the difference in participant experiences between Push ball (left) and Launch ball (right) during hand movements. **f** Distance moved over time for a Push ball and Launch ball trial (points represent individual mouse position samples). Reaction time is the interval between ball appearance and click, stationary time is the duration between the click and movement onset (orange to green), and movement time is from movement onset to trajectory end (green to purple). Push ball trials show longer movement times and a change in velocity as the participant integrates online feedback.

Each game consisted of four sequential blocks: baseline, learning, error clamp, and washout (Fig. 1b). During baseline and washout, there was no perturbation. During learning, a 30-degree clockwise visuomotor rotation was gradually introduced over 60 trials and then maintained for an additional 30 trials. To succeed at the task the participant would need to counteract the visual rotation perturbation (see Fig. 1b, 2^nd^ panel in learning). The error clamp block was used to assess retention of adaptation in the absence of error signals^25,26^. During the error clamp block, we artificially controlled the rotational visual error so that each trial was successful independent of the mouse movement. The mouse movement was recorded, and the ball moved at the same speed as the child in the y direction but was displayed as moving nearly straight towards the target (with a small, random rotation of −2 to 2 degrees added each trial to mimic natural trial-to-trial variation).

### Push ball

Four hundred and ninety-four participants completed the Push ball task (demographic information and age group breakdown in Supplementary Table 1). Participants took 19.4 ± 9.4 minutes (mean ± SD) to complete the game with completion time decreasing with age (F_1492_ = 118.5, *p* < 0.001, adjusted r^2^ = 0.19). Sex distribution was similar across the age groups (F_7486_ = 1.22, *p* = 0.29).

**Fig. 2:**
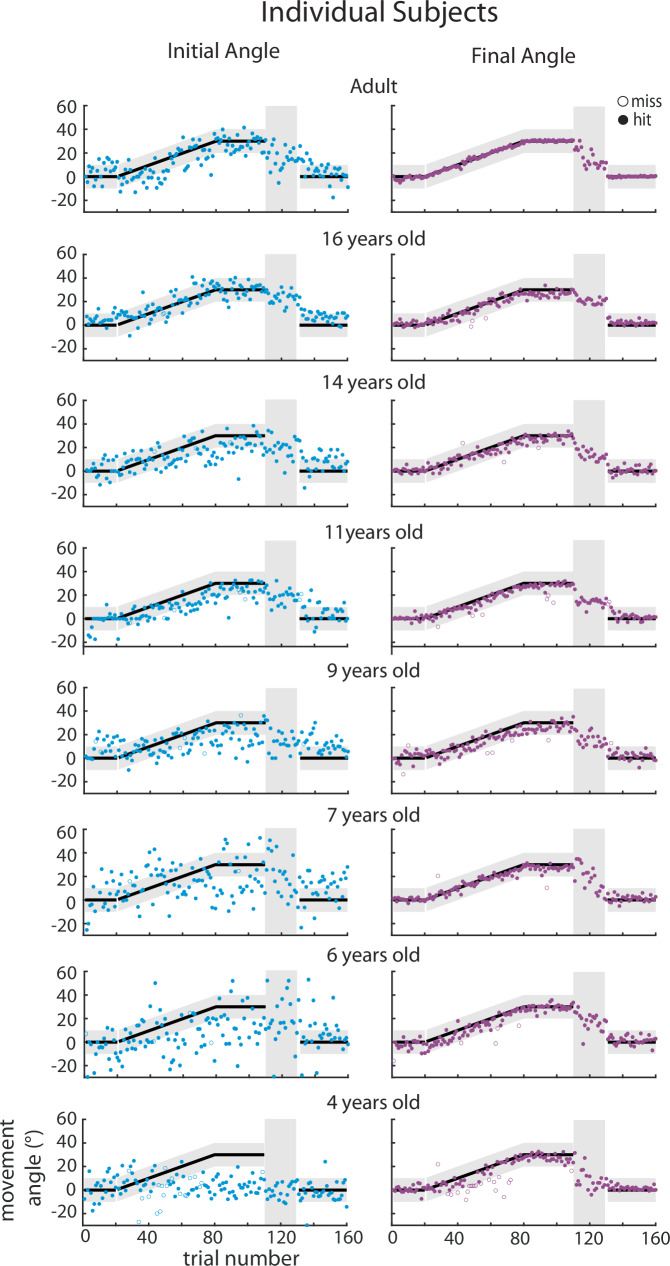
Trial-by-trial data of the initial and final angles for Push ball by age bin. Trial-by-trial data of the initial angle (adaptation, blue) and final angle (overall performance, purple) for Push ball by age bin. Individual participant data are shown for each age bin for the initial angle (first column) and final angle (second column) with successful trials in filled circles and unsuccessful trials in open circle. Black solid line with gray shaded region demonstrates when participants would be successful at hitting the play button.

**Fig. 3:**
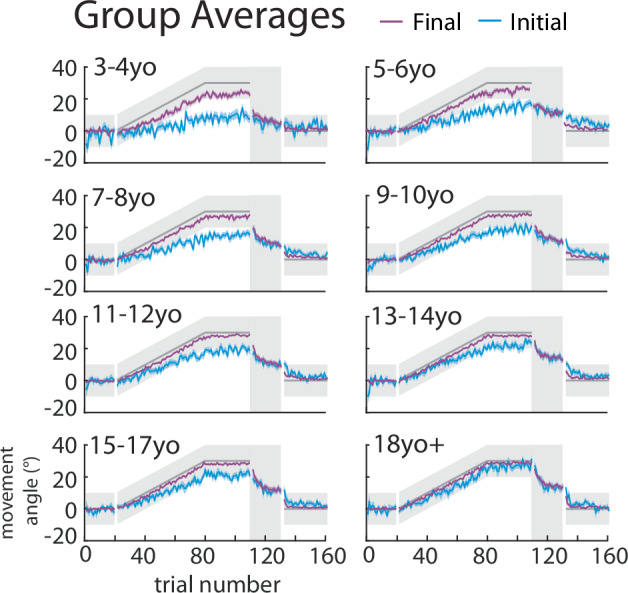
Group averages for Push ball by age bin. The mean ± standard error (s.e.) across participants in each age bin for the initial (blue) and final (purple) angle overlaid.

To assess feedforward adaptation, we used the initial direction (angle) of each movement (Fig. 1d, blue) and to assess overall task performance we used the final angle at the end of the movement (Fig. 1d, purple). Figure 2 shows individual example participants’ data and Fig. 3 shows group data binned by age for these measures. The group data shows that, on average, all age groups were able to successfully hit the target throughout all blocks of the experiment (final angle, purple falls in the gray target zone). In contrast, the initial angle fell outside the angle required for success (that is if this angle had been maintained throughout the entire movement) for the younger children, and was only, on average, within the target for children older than 13 years. This finding suggests that the younger children may be using a feedback strategy to correct for a deficit in adaptation. During the error clamp phase (trials 110–130), the initial and final angle were similar within each group suggesting that the lack of error led to little correction from the initial angle. In addition, in the absence of error in this block these measures partially decayed.

**Fig. 4:**
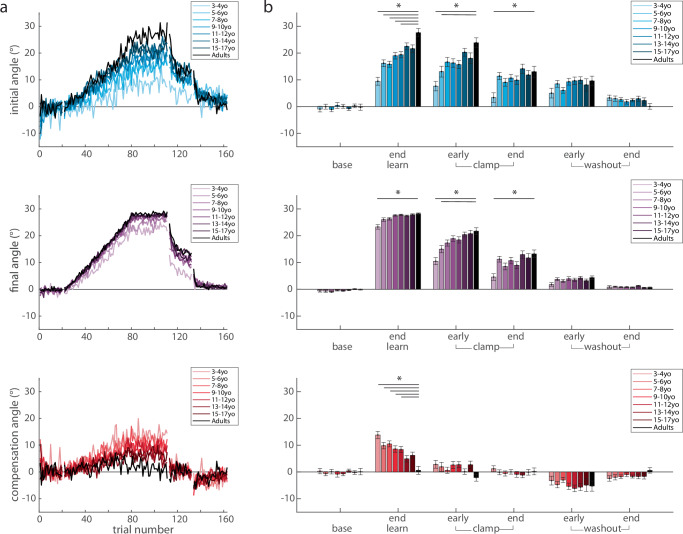
Push ball overlaid trial-by-trial group averages and experimental epochs by age bin. Trial-by-trial group averages (**a**) and epochs (**b**) of the initial angle, final, and compensation angle by age bin throughout the experimental paradigm. Mean ± s.e. are shown in the epoch bar plots. Younger children are lighter colors compared to adolescents in darker colors and adults are in black. Statistically significant differences when age groups are compared to adults are shown by an asterisk (*p* < 0.05).

As a measure of feedback control (i.e. steering the ball^27^, we calculated the compensation angle as the difference between the final and initial angle (compensation angle, Fig. 1d red). Figure 4 shows the three measures across the experiment, split by age groups. Initial angle (top) shows a graded increase with age in the learning block, whereas the final angle (middle) shows less change with age (Fig. 4a, top and middle). This leads to a gradient in the compensation angle (bottom) which decreases with age (Fig. 4a, bottom). That is younger children demonstrate more online feedback to hit the target. Importantly, the initial angle does not return to baseline during the error clamp period, indicating retention, and then decays gradually during washout.

To quantify these effects, we calculated the three measures over different phases of the experiment (Fig. 4b) and performed separate repeated measures ANOVAs for each measure by epoch x age-group. For the initial angle, there was a significant effect for epoch (F_4.23,2056_ = 320.6, *p* < 0.001) and age-group (F_7486_ = 6.12, *p* < 0.001), and interaction (F_29.6,2056_ = 4.54, *p* < 0.001) (Fig. 4b, top). Importantly, there were no differences in baseline across age-groups, indicating all children and adults started with similar behavior. At the end of learning, post hoc analysis revealed that when compared to the adult group, the 3–4yo (*p* < 0.001), 5–6yo (*p* < 0.001), 7–8yo (*p* < 0.001), 9–10yo (*p* = 0.004), 11–12yo (*p* = 0.01) all were statistically different, whereas the 13–14yo (*p* = 0.76) and 15–17yo (*p* = 0.47) groups were not. This result indicates that adaptation matures or appears “adult-like” around adolescence. In early clamp, the initial angle was significantly different for the 3–4yo (*p* < 0.001) and 5–6yo (*p* = 0.01) when compared to the adults, indicating significantly smaller after-effects in the youngest age groups. The end of clamp was also different between the 3–4yo and the adults (*p* = 0.01). Other epochs demonstrated no differences when compared to adults.

An important hallmark of adaptation due sensorimotor recalibration is the presence of an after-effect when the perturbation is removed. This after-effect requires many trials to return to the unperturbed state. To confirm the presence of after-effects, we investigated post-hoc effects of the initial angle between the clamp and washout blocks when compared to baseline. Collapsing across all groups, we found significant differences between early clamp and baseline (*p* < 0.001), end clamp and baseline (*p* < 0.001), early washout and baseline (*p* < 0.001), and even the end washout and baseline (*p* < 0.001). We then tested the effect of age group, and found similar results in the clamp block, and a smaller age-related effect in the washout block. Figure 4b (top panel) shows that the older age groups tended to return back to baseline initial angles by the end of washout, whereas younger children did not. When compared to baseline, the youngest children’s (3–4yo) initial angle was significantly larger in the early clamp (*p* < 0.001), early washout (*p* = 0.001), and late washout (*p* = 0.003), indicating that the learned pattern was stored and persisted well after the perturbation was removed. In the older groups, we found significant differences between baseline with early and late clamp (all *p* < 0.001) and early washout (all *p* < 0.002). By the end of washout, the initial angle did not reach significance for most of the age groups (5–6yo, *p* = 0.08; 7–8yo, *p* = 0.01; 9–10yo, *p* = 1; 11–12yo, *p* = 1; 13–14yo, *p* = 0.03; 15–17yo, *p* = 1, adults, *p* = 1). In sum, we found that when compared to baseline, all age groups demonstrated after-effects in the clamp and washout blocks, with the older children and adults returning back to baseline performance by the end of washout while the younger children did not.

For the final angle, there was a significant effect for epoch (F_2.86,1391_ = 1525, *p* < 0.001), age-group (F_7486_ = 8.64, *p* < 0.001), and interaction (F_20,1391_ = 4.42, *p* < 0.001) (Fig. 4b, middle). Similar to the initial angle, there were no differences in baseline final angle across age-groups. Although on average all age-groups were able to successfully hit the target (final angle between 20 and 40 degrees), the youngest group (3–4yo) had significantly smaller final angles at the end of learning when compared to the adults (*p* < 0.001). In early clamp, as expected, the final angle was similar to the initial angle. The final angle was significantly different for the 3–4yo (*p* < 0.001) and 5–6yo (*p* = 0.04) when compared to the adults. At the end of clamp, the 3–4yo were statistically different than the adults (*p* = 0.004), which indicates decreased retention for this youngest group. There were no statistically significant differences in washout when compared to the adult group.

We found that the use of online feedback control, as measured by the compensation angle, allowed younger children to be successful at the task even if they did not fully adapt their initial angle to account for the perturbation. For the compensation angle, there was a significant effect for epoch (F_4.52,2197_ = 117, *p* < 0.001), epoch x age-group (F_31.6,2197_ = 2.36, *p* < 0.001), but not for age-group (F_7486_ = 1.62, *p* = 0.13) (Fig. 4b, red, bottom row). Again, baseline behavior was similar across age groups. At the end of learning, post hoc analysis revealed that the compensation angle was significantly different for the 3–4yo (*p* < 0.001) and 5–6yo (*p* < 0.001), 7–8yo (*p* < 0.001), 9–10yo (*p* = 0.007), 11–12yo (*p* = 0.02) when compared to the adults. This finding shows that up until adolescence, children are using more online feedback to successfully counteract the perturbation. Note that during the clamp, compensation angles are close to zero across all age-groups. This is indicative of participants making generally straight-line movements in the clamp phase since they were not given any feedback of errors as they moved (Fig. 4b, compensation angle, early and end clamp epochs). Once again, when feedback becomes veridical again in washout, we see that participants *compensate in the opposite direction*, revealing retention of their adapted state that must be ‘unlearned’ in the washout (Fig. 4b, compensation angle, early WO). There were no differences in the clamp or washout across groups.

Prior studies have suggested that there are small sex-based differences in motor learning and control^28,29^. However, we did not find an effect of sex on either the initial (F_1478_ = 0.4, *p* = 0.53) or final angle (F_1478_ = 1.6, *p* = 0.20). There was an effect of device – those completing the task with a touchscreen had the smallest change in initial and final angles, followed by those that completed it with a mouse, and then those who used a trackpad. We did not find any interaction of the device with age-group or epoch, signifying that the effect of device was similar across all age groups and throughout all blocks of the experiment. There was also a small effect of handedness (left-handed with slightly larger final angles), but only 32 participants were left-handed. Further details and analyses on the effects of sex, handedness, and device are outlined in the Supplementary Results.

**Fig. 5:**
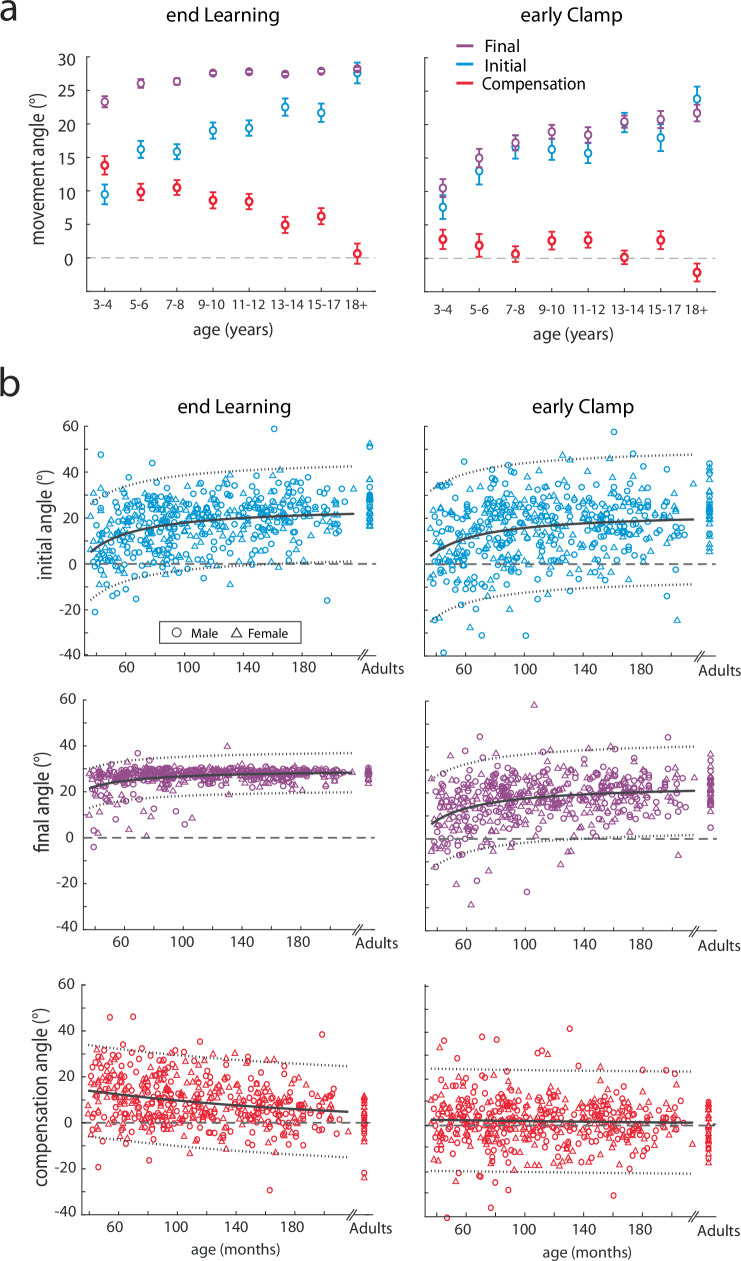
Group averages and scatterplot of individual subject performance at the end of learning and early clamp. **a** Initial, final, and compensation angle by age bin (mean ± s.e.) overlaid. Note how at the end of learning younger children have larger compensation angles than initial angles whereas adolescents have larger initial angles and minimal compensation. In the early clamp, the initial and final angles by age bin are very similar with compensation angles around zero indicating that the clamp block reflects the aftereffect of adaptation. **b** Average learning and retention of the initial, final, and compensation angle for individual subjects in Push ball by age. Males (circles) and females (triangles) are shown with adults displayed on the right-hand side of the plot. Inverse (initial and final angle) and exponential (compensation angle) fits (solid gray line) and the 95% confidence intervals (dotted gray lines) are also shown for the children. Adults were not included in the fits. For the initial angle at the end of learning the model fit the parameters were: a = 25.16 (22.99, 27.34), b = −720.9 (−899.8, −541.8) with an adjusted R^2^ = 0.12. For the final angle at the end of learning the model fit the parameters were: a = 29.7 (28.8, 30.6), b = −295.3 (−369.1, −221.6) with an adjusted R^2^ = 0.12. For the compensation angle at the end of learning the model fit parameters were: a = 17.35 (13.3, 21.4), b = −0.006 (−0.008, −0.004) with an adjusted R^2^ = 0.06. For the early clamp, the initial angle fit was a = 22.68 (19.71, 25.65), b = −692.7 (−937, −448.3) with an adjusted R^2^ = 0.06 and the final angle fit was a = 23.88 (21.85, 25.9), b = −629.6 (−796.3, −462.8) with an adjusted R^2^ = 0.10. For the early clamp, the compensation angle confidence intervals crossed zero and were: a = 2.849 (−0.89, 6.6), b = −0.004 (−0.02, 0.009) with an adjusted R^2^ = 0. Note that the compensation angle in early clamp is essentially flat across ages.

In sum, there appears to be a developmental tradeoff in the use of adaptation and online feedback control. Younger participants adapted their initial angle less and relied on online feedback control more to be successful (Fig. 5a left, end learning). The 3–4yo children use more online feedback than adaptation. This pattern shifted gradually with the age of the participants up until adolescence, when 13–14yos primarily utilized adaptation with limited online feedback control, similar to the adults. Importantly, there was good overall performance across all age groups – even children as young as 3–4yo were able to hit the target.

To quantify the gradual maturation of adaptation, overall performance, and compensation during this task, we fit these measures at the end of learning and beginning of the clamp phase as a function of age. We chose a two-parameter nonlinear function (equivalent to a fit in which the measures are a linear function of the reciprocal of age). Adults were excluded from the fitting analysis but are shown in Fig. 5b for reference. Figure 5b shows that the initial angle (reflecting adaptation) has a similar age-dependent pattern when measured at the end of learning versus early in the clamp phase. We correlated the initial angle at the end of learning with the initial angle in the early clamp and found a significant linear relationship very close to the unity line (F_1492_ = 282.5, *p* < 0.001, adjusted r^2^ = 0.36) (Supplementary Fig. 9C). This suggests that the adapted pattern was stored. Also note that the initial and final angle behavior looks very similar in the early clamp (Fig. 5 right panels), and they look different at the end of learning (Fig. 5 left panels), reinforcing that the error clamp trials were effective at measuring retention of the feedforward adaptation learning mechanism. In addition, we found that the compensation angle decreases with age (Fig. 5b red) at the end of learning and is essentially flat during early clamp. This once again reinforces the idea that there is a developmental tradeoff between adaptation and online feedback that changes across age even when looking at individual subject behavior.

Mature motor control not only requires accuracy but also precision in movement. As such, we also wanted to assess how variability of the initial and final angle changed with age. These data are shown in Supplementary Fig. 1 with statistics in the legend; adults were excluded from the fitting analysis but are shown for reference. Overall, we found that the variability of both the initial angle and final angle decrease with age. As expected, the initial angle is more variable than the final angle throughout childhood. Additionally, the variability of the initial angle takes longer to decrease compared to the final angle (initial angle end learning < initial angle baseline < final angle end learning < final angle baseline).

We then investigated whether younger children’s movement trajectories reflect their use of online feedback control compared to older children. We expected that younger children would make curved paths and use corrective movements, whereas older children would not. First, we calculated the path length ratio, as a measure of path straightness, defined as the ball’s path length divided by the distance between the first and last point of the trajectory^30–33^. We found that there was a significant effect of age-group on the path length ratio (F_7486_ = 17.53, *p* < 0.001, Supplementary Fig. 3a). Excluding adults from the regression analyses, we found a significant effect of age (without grouping) on path length ratio (β = −0.003, 95% CI: −0.003 to −0.002, F_1462_ = 79.0, *p* < 0.001, adjusted r^2^ = 0.14). Older children had straighter paths than younger children, suggesting that they may be using less online feedback control.

**Fig. 6:**
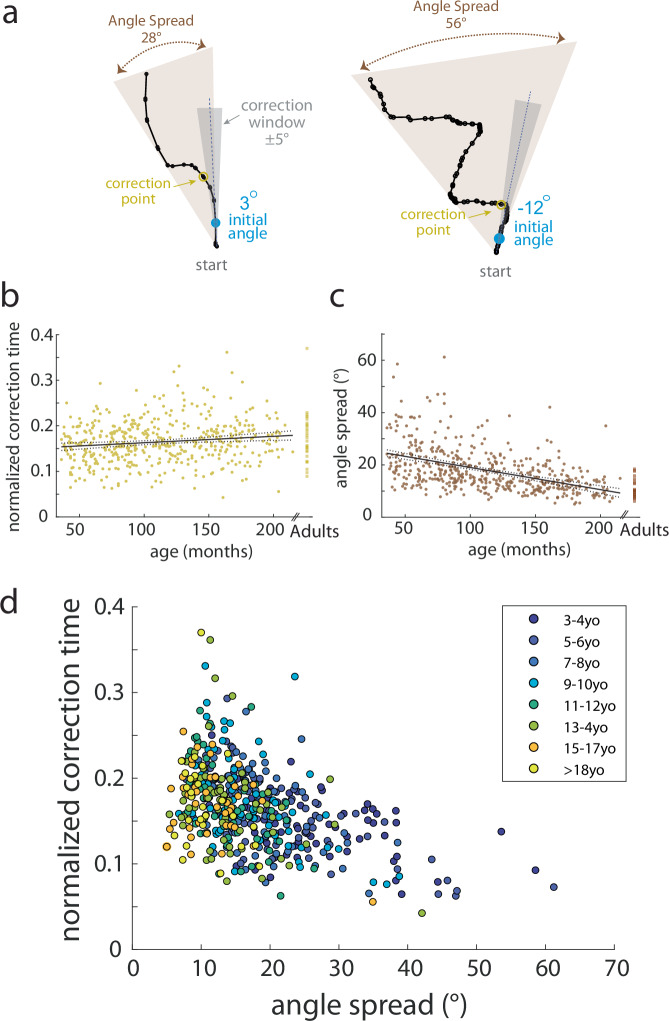
Push ball trajectory analysis by age. **a** Example trajectories (black line) demonstrating correction point (yellow) and angle spread calculations (brown) to capture trajectory behavior of participants. The correction window (gray zone) is determined as ±5° from the initial angle (blue). Note how the path length ratio for some children can be larger due to curvature or due to zigzag or curly trajectories. **b** Scatterplot of the normalized correction time by age for children with adult data shown off to the right. Smaller values indicate earlier correction in the movement time. Linear regression (solid gray line) with 95% CI (dotted gray line) are shown. Older children correct later in the movement. **c** Scatterplot of angle spread by age for children with adult data shown off to the right. Smaller values indicate smaller angle spread. Linear regression (solid gray line) with 95% CI (dotted gray line) are shown. Older children have a smaller angle spread to their trajectories. **d** Relationship between the normalized correction time and angle spread as indicated by age bin (younger = purple, older = yellow). Younger children have a larger angle spread in their trajectories and correct earlier in their movement.

Participants’ longer path length ratios could be due to different patterns of movement. For example, children could make a single, curved movement path or show repeated curves and zigzags (Fig. 6a), the latter of which might reflect less accurate motor control. To better distinguish these behavioral differences, we analyzed spatial correction parameters. We quantified where the first correction occurred in the movement. We defined this as the first time the path deviated by more than 5 degrees from the initial angle (Fig. 6a, dark gray shows the correction window and yellow is the correction point). The *normalized correction time* was the time to the correction point divided by the movement time. We normalized the time to correction because =younger children had longer movement times. In other words, this parameter identifies how far into the movement participants started to correct.

We also quantified angle spread as the absolute difference between the minimum and maximum movement angle across the movement (Fig. 6a, light gray). Similar to the path length ratio, we found a significant effect of age on the normalized correction time, which demonstrates that older children corrected later in the movement than younger children (F_1462_ = 9.3, *p* = 0.002, adjusted r^2^ = 0.018) (Fig. 6b). Younger children had larger angle spread indicating their trajectories covered more of the game space and had more curvature (F_1462_ = 128.6, *p* < 0.001, adjusted r^2^ = 0.22) (Fig. 6c). Using a multiple regression, we found significant effects of both age (*p* = 0.03) and angle spread (*p* < 0.001) on the normalized correction time (F_2461_ = 69.5, *p* < 0.001). Taken together, as seen in Fig. 6d, younger children had more curved trajectories which required *corrections earlier in their movement*, suggesting a reliance on online feedback, confirming the results reported above with our primary parameters.

Although we used spatial parameters as our main outcome variables due to irregular sampling rates of participants’ home computers, we did analyze timing parameters. Overall, we found that younger children tended to move slower, resulting in longer reaction times, stationary times, and movement times compared to adolescents and adults (see Supplementary results for further details). As such, we also found that the time to initial angle calculation (IA_time_) was slightly longer for younger kids (e.g., IA_time_ of 3-4yo: 445 ± 51 ms) compared to adults (115 ± 96 ms). Despite the initial angle being calculated later in the movement for younger children, we still find that younger children adapted less. If the initial angle calculation in the younger groups did include some online feedback corrections, their true adaptation ability would be even less than what is observed here.

To investigate the influence of age, sex, and baseline variability on learning, we performed stepwise linear regressions. Age was the strongest predictor of both adaptation (i.e., initial angle) and overall performance (i.e., final angle) at the end of learning. For adaptation, variability of the final angle at baseline, indicative of general of motor control, was a secondary predictor. Further findings are presented in the Supplementary results.

### Launch ball

The Launch ball task was designed to assess whether younger children’s use of online feedback reflected a preference or a reliance. In other words, did younger children choose to use online feedback even if they could use adaptation (a preference) or did they need to use online feedback to complete the task because they were unable to rely on adaptation (a reliance)? We analyzed data from 162 participants (see Methods for details), aged 5 and older. It took participants 20.8 ± 7.3 min (mean ± SD) to play the game which was similar to the game play duration in the Push ball task. Also similar was the inverse association between game duration and age (F_1160_ = 21.2, *p* < 0.001, adjusted r^2^ = 0.11). Additional participant characteristics are shown in Supplementary Table 1.

In Launch ball, participants could not use online feedback to correct movements during a trial; participants could only use an adaptation learning mechanism. To play the game, participants moved forward in the direction they wanted the ball to move with the mouse button depressed. The ball was only launched and started to move when they released the button. The ball then moved in a straight-line path passing through the location (or rotated location during learning) where the mouse was released (Fig. 1d, Launch ball). After the trial was complete (i.e., the ball hit or missed the target), the ball reappeared at the start and the participant was instructed to click the ball to start the next trial.

The launch angle was computed at the time the participant released the mouse button (see Methods). Once the ball was launched, participants could not steer the ball (Fig. 1e). This made the launch angle similar to the initial angle in the Push Ball game (and the final angle is now the same as the launch angle). Examples of individual participants’ trial-by-trial behavior of the launch angle and their hand trajectory data are shown in Supplementary Figure 4 for the entire experiment. Note that younger children do not change the launch angle appreciably during the learning block, whereas the older children do. Also note that the mouse trajectories are relatively straight since visual feedback was not available to the participants as they were moving.

**Fig. 7:**
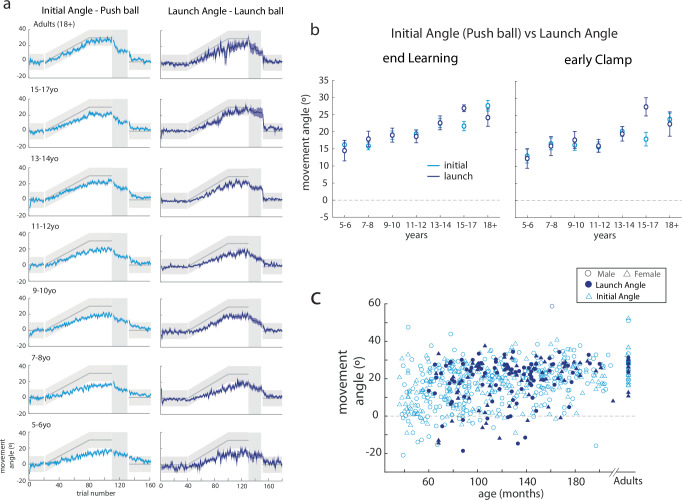
Comparison between Launch ball and Push ball results. **a** Trial-by-trial data (mean ± s.e.) of the initial angle in Push ball compared to the launch angle in Launch ball by age bin for children aged 5 and above. Both parameters capture adaptation in the two tasks. Note how similar performance is on the two tasks. There is more variability across participants in the Launch angle, but the sample side was smaller in this task compared to Push ball. **b** Comparison of the initial angle in Push ball to launch angle in Launch ball by age bin at the end of learning and early clamp. There was no difference in the task across ages. **c** Scatterplot of individual participant’s initial angle (Push ball) (open light blue symbols) and launch angle (Launch ball) (filled dark blue symptoms) with males in circles and females in triangles.

Figure 7a shows trial-by-trial data of the Push ball initial angle and the Launch ball launch angle binned by age. Note that the pattern of behavior is similar between the two tasks, across the age groups. Here again, younger children do not change their initial angle or launch angle much, whereas older children adapt to the gradually imposed perturbation. Figure 7b shows behavior on the two tasks, binned by age group. We used repeated measures ANOVA to compare epochs of the experiment, with task and age-group as main effects. For this analysis, we included Push ball participants who were aged 5 years and older, since we did not test Launch ball on 3-4yo children. There was no effect of task (F_1571_ = 0.007, *p* = 0.94) nor a task x age-group interaction (F_6571_ = 0.95, *p* = 0.46). As expected, there was a significant effect of age-group (F_6571_ = 2.8, *p* = 0.01). Figure 7c shows a scatter plot of Push ball initial angles and Launch ball launch angles at the end of the learning block, as a function of age. Note that the two groups overlap one another, again suggesting that the behavior is similar between tasks, even at an individual subject level. Two important conclusions come from this analysis. First, this finding confirmed that our initial angle parameter accurately captures adaptation (i.e., feedforward or predictive behavior). Second, we found that younger children must rely on online feedback to successfully complete the task due to immature or developing adaptation motor learning mechanisms. Younger children did not adapt more in Launch ball – instead, they were just less successful at the task (i.e., they miss the target more).

After-effects in the launch angle for Launch ball were also similar to the initial angle in Push ball. To summarize, we found that when compared to baseline, all age groups demonstrated after-effects in the clamp. Younger children then had persistent after-effects until the end of washout, whereas adults returned to baseline early in washout (Supplementary Fig. 5 and see Supplementary results “Launch ball” for further details).

Participants make straighter movements in Launch ball compared to Push ball, as reflected by the path length ratio (Supplementary Fig. 3b). There was no benefit to curved trajectories in Launch ball. For example, compare the 6-year and 7-year old’s “Learning” trajectories in Launch ball (Supplementary Fig. 4) to the 7-year old’s “Learning” trajectories in Push ball (Supplementary Figure 6). Path length ratio was similar across age groups in Launch ball (F_6155_ = 1.01, *p* = 0.42). In contrast, in Push ball, we found a significant effect of age-group on the path length ratio—younger children had longer path length ratios than older adolescents and adults. This is consistent with the younger children’s use of visual online feedback corrections to steer the ball and successfully hit the target.

Supplementary Fig. 7 shows the timing parameters for Launch ball across age groups. Movement time was not statistically different across age groups (F_6155_ = 1.61, *p* = 0.15) in Launch ball, which is different than what was observed in Push ball (younger children took longer). Older participants did have shorter reaction times (F_6155_ = 5.86, *p* < 0.001) in Launch ball. Also, the youngest children (5–6yo) had slightly longer stationary times than the other groups (F_6155_ = 5.34, *p* < 0.001). Thus, younger participants playing Push ball slowed their movement and made curved paths, both of which support their use of online feedback corrections.

In summary, comparing the age-based results on Launch ball and Push ball suggests that younger children have underdeveloped adaptation motor learning mechanisms. As a result, children learn to be successful at reaching targets with the compensation of on-the-fly online feedback corrections. However, the online feedback corrections seen in younger children took longer than what would be expected from visual processing time delays alone. It may be that younger children needed more time to use visual feedback to decide where to steer the movement. In contrast, older children may have developed the ability to make these decisions more quickly. When younger children need to rely only on the adaptation learning mechanism, they can make faster movements, but are less successful.

### Percentile curves

**Fig. 8:**
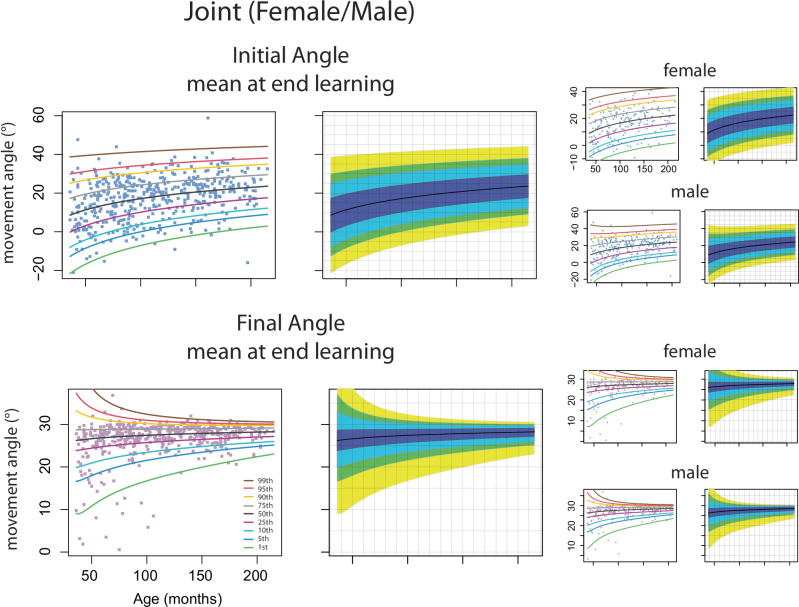
Percentile curve estimation for mean of the initial and final angle. Percentile curve estimation for mean of the initial and final angle at the end of learning of all children and separated by female and male from Push ball data. Adults are not included. Data from individual children are shown in the filled boxes with the percentile estimates shown in the solid lines. The percentile curve estimates are also demonstrated in the fan plot without data shown to better show the pattern of age-based effects.

A goal of this project was to construct percentile curves of adaptation and overall performance as a function of age across children, aged 3–17. This is important as it provides a quantitative summary of developmental patterns of motor processes that can be used as a reference by researchers who study motor learning. We created smooth percentile curves for the amount of adaptation (mean initial angle at the end of learning epoch) and overall performance (mean final angle at the end of learning epoch, reflecting adaptation + online feedback corrections). We also show percentile curves for the standard deviations (precision) of these measures. Percentile curves were calculated for the entire sample, and then broken down by sex. Figure 8 and Supplementary Table 2 show estimated 1%, 5%, 10%, 25%, 50%, 75%, 90%, 95%, 99% percentile curves. The adaptation measure (i.e., initial angle) increased gradually across age for the overall group, with males and females showing similar patterns. Thus, adaptation ability was immature in younger children and gradually matured with age. The final angle sharpened across age, indicating that the individual participant’s overall performance was more variable in younger children than older children. Supplementary Fig. 8 shows the percentile curves for the standard deviation of the initial and final angles at the end of learning. The standard deviation is larger for the initial angle vs. final angle across ages. There is an age-dependent reduction in the standard deviation for both initial and final angles, although it is more protracted for the initial angle.

## Discussion

Motor learning is the process of acquiring or altering a movement through practice^34^. It enables individuals to develop, refine, and adapt the motor skills needed to perform a wide range of activities from basic movements (e.g., grasping a ball) to complex, specialized tasks (e.g., playing the piano). Though motor learning is pivotal to the development of motor skills throughout childhood, very little is known about the developmental timeline of motor learning mechanisms that allow for flexibility in movement.

We examined a large, cross-sectional cohort of 3–17-year-old children and adults as they performed error-based visuomotor rotation adaptation tasks at their home computers. Children used a mouse to try to control a ball to hit a target which would cause a video clip to play. Unbeknownst to the participants, a perturbation was gradually introduced to create a mismatch between where the participants’ mouse moved and where the ball went on the screen. This perturbation resulted in children learning a new movement pattern. We tested two tasks: one in which children could be successful either using feedforward adaptation mechanisms or online feedback corrections (Push ball) and a second where children could only use adaptation mechanisms (Launch ball).

We found a developmental trajectory of motor learning mechanisms using the Push ball task, with younger children adapting less than older children. Perhaps surprisingly, our data suggest that children up until adolescence (e.g., ~13–14 years old) still demonstrate immature adaptation, even in this upper extremity task. This protracted developmental trajectory closely parallels previous work from our group demonstrating that walking adaptation was not mature or “adult-like” until adolescence (e.g., ≥13 years)^18^. Yet the current work differs from prior studies of reaching adaptation to force fields which have shown that development is mature or adult-like at an earlier age (~7-10yo)^19,20^. The measures taken in prior studies were across the entire reaching movement, which is more similar to our final angle measure and includes the entire movement. When we analyze the final angle, we find that younger children (5–6-year-olds) perform similarly to adults (Fig. 4). It is only once we decompose the final angle into the initial angle, representing adaptation, and compensation angle, representing online feedback, that we see a more accurate representation of the tradeoff between adaptation and online feedback throughout development. This interpretation is also consistent with other work showing that, even in the absence of perturbations, young children depend more on online feedback during repeated rapid aiming movements to a stationary target^21–23^. Children younger than 9 years of age tend to plan only the initial portion of their movement and then use proprioceptive feedback to make corrections during execution^21,22^. In our study, younger children had more curved trajectories and made corrections earlier in their movement compared to adolescents and adults (Fig. 6d). It is important to note that our results do not suggest that older children and adults cannot use online feedback in reaching movements. Since they were better at adapting their initial angle in response to the perturbation, they did not need to use online feedback to the same degree as younger children. In sum, we found that children younger than 14 years old were less likely to use adaptation learning mechanisms to overcome a visuomotor rotation perturbation. Younger children were able to compensate for less adaptation by relying on online feedback to achieve success.

We used a separate experiment to ask if our Push ball task encouraged younger children to use online feedback control even if they could adapt more. Results from our Launch ball task showed that younger children’s reliance on feedback control is not just a preference. When faced with a situation where they had to use adaptation to succeed at the task, younger children were unable to learn and missed the target (Launch ball, Fig. 7). This finding leads us to believe that, on average, adaptation motor learning mechanisms are immature and still developing until early adolescence.

One difference to consider is that most reaching tasks that have been studied used an abrupt perturbation and learning rates to assess age-dependent effects^18–20^. Here we chose to use a gradual perturbation because, when pilot testing the youngest children, they became frustrated with the abrupt perturbation. They thought that there was a problem with the game, causing them to quit prematurely. The only way we could reliably test children under age 6 was to use a gradual perturbation. It should be noted, however, that with the gradual perturbation we cannot directly assess learning rates, so instead we quantified learning and retention magnitudes.

Additionally, the gradual perturbation is more likely to bias participants to utilize adaptation to recalibrate their sensorimotor mapping, as opposed to using a more explicit strategy^35^. While we cannot exclude that participants used some component of explicit strategy during the learning, the presence and persistence of after-effects throughout the clamp and washout blocks suggests that adaptation was a significant component of the feedforward control. The gradual decay during the clamp phase has been previously demonstrated in adults, and is thought to represent the gradual unlearning of the implicit recalibration process due to the lack of visual error signals^36^. We acknowledge that gradual decay during clamp could theoretically be due to forgetting of an explicit strategy but think that the washout behavior helps to differentiate between these two mechanisms. If an explicit strategy was the primary driver for the initial angle changes, we would expect a return to baseline within 1–2 trials of washout (where participants see large errors in the opposite direction)^37^. Instead, we see that the after-effects persist longer in washout, even in adults. We find that the youngest children still demonstrate after-effects at the end of washout^18,38^. It may be that the contribution of an explicit strategy changes across development, or that the implicit learning and forgetting rates are simply slower in younger children. These ideas could be investigated in future studies by providing an additional target that would compensate for the perturbation during learning, similar to the paradigm in Mazzoni and Krakauer^37^ or by asking participants to report their aiming location throughout or at the end of the experiment^39^.

One hypothesis is that age-related adaptation ability is dependent on protracted development of the cerebellum throughout childhood. Cerebellar damage impairs adaptation across reaching^9,13^, walking^8^, eye movements^12,40^, and postural control^41^. Neuroimaging studies that examine the volumes of different regions provide some guidance in thinking about maturation of neural structures important for motor learning and motor control. Regions that reach peak volume earlier are thought to mature earlier. Adaptation and online feedback rely on different neural structures. Adaptation correlates with activity between the cerebellar cortex and frontal motor areas (M1 and SMA)^42^. Integration of online visual feedback during reaching requires network activity of visual cortex with parietal, dorsal premotor (PMd), and motor cortical areas^43–51^. Primary motor and sensory cortices peak in childhood (age 5–10)^52^ whereas the cerebellum^53,54^ peaks later (age 12–16). These imaging studies provide some neuroanatomical evidence for the developmental trajectory of adaptation and online feedback seen in our behavioral study.

Another hypothesis is that other neural networks limit adaptation ability during this visuomotor rotation task. Cognitive factors can influence adaptation in adults^55–61^. Studies in adults also show that working memory^57,62,63^, and in particular spatial working memory^58,59^, can influence visuomotor adaptation. In particular, a decline in spatial working memory contributes to age-related declines in visuomotor learning^58^. Neurocognitive testing has revealed that working memory is still developing until 15 years of age, when it reaches adult-like behavior^64,65^. Thus, it is possible that cognitive development might also contribute to age-related effects and differential performance of children on the task within an age group.

There are not only age-related effects on the accuracy of children during this task (i.e., mean values) but also in the precision or variability (i.e., standard deviation). To be truly skilled at a motor task, a child needs to be accurate and precise. In other words, their movements need to be reproducible. Throughout infancy and early childhood, movements are variable. This variability allows the nervous system to try out many functional options and is important for development^66^, so much so that limited movement variability during infancy has been shown to be a predictor of later motor impairment and cerebral palsy^67^. As a child ages, too much variability can be detrimental for motor control. If a child can only rarely get the spoon in their mouth, they would not be able to feed themselves efficiently and gain independence. In line with these ideas, we find that adaptation, as measured by the initial angle, is more variable than the overall performance, as measured by the final angle, across ages, and that variability decreases with increasing age (Supplementary Fig. 1). Interestingly, baseline standard deviation of the final angle, which is a proxy for motor precision, was the strongest predictor of the overall performance at the end of learning. It also played a role in how well the participant adapted, but age was the primary driver in adaptation learning.

Contrary to a recent paper^68^, we found no sex-related differences in adaptation throughout childhood (i.e., in the initial angle). Tsay et al. 2024 found that sex affected the early part of an abrupt adaptation thought to rely more on explicit strategies^68^. With our gradual perturbation task, it’s possible that explicit strategies were less contributory. Other factors such as video game/computer usage or enjoyment might contribute to variability seen across participants, as was shown in Tsay et al. ^68^, but were not assessed in this data set. This study was not designed to look at specific race, ethnicity, or socioeconomic status related effects on adaptation, but they could be investigated in future studies using this type of novel methodology.

We established percentile curves for the initial and final angle at the end of learning to provide a quantitative report of how age affected adaptation and overall performance. These percentile curves are designed to be used as a reference, indicating how a child performed compared to other children aged 3–17 years on the Push ball task^69^. For example, Supplementary Fig. 10 shows different patterns when comparing two 4-year-olds and two 10-year-olds. The 4-year-old participants (blue triangle, pink square) showed differences in adaptation and overall performance. The pink square participant adapted less and had a smaller change in their final angle (they were below the median on both parameters). Conversely the blue triangle participant adapted and changed their final angle more than other 4-year-olds (50–75^th^ percentile). The blue triangle participant also showed more variability in their initial angle compared to that of the pink square participant. The 10-year-old participants (orange diamond and green circle) illustrate different patterns. The orange diamond participant adapted their initial angle more than the green circle participant, yet the final angle performance for both children was comparable. These references are a critical first step in the development of growth curves of adaptation; they tell us about the variability across children at different ages and how an individual child performs compared to age-matched peers. But it is important to note that these reference percentile curves should not yet be used as standards, adding value judgements and detailing how a child *should* adapt and perform^69^. As part of future studies, we hope to investigate neurocognitive factors that may influence or explain an individual child’s adaptation and overall performance. In the future, we intend to take the next steps including studying longitudinal cohorts, correlating these measures with clinical measures, and investigating behavior of children with pediatric disorders to better inform patterns of abnormal development.

An important challenge is to understand the normal trajectory of motor learning abilities so that we can begin to interpret, understand, and ameliorate the effects of damage to the nervous system during development. Here we have examined a large, cross-sectional cohort of 3–17-year-old children and young adults as they performed an error-based visuomotor rotation task. We were able to describe a behavioral developmental trajectory of adaptation in typically developing children. This study has important implications to better understanding how motor learning mechanisms develop throughout childhood and the ability to inform future rehabilitation treatments to utilize mechanisms that are online and available in children.

## Methods

### Participants

All participants were recruited from the Johns Hopkins University community through an online announcement portal, the greater Baltimore Maryland area through Research Match, and nationwide through the online platform Look it which merged with Children Helping Science in 2023^70^^,71^. Participants were screened to rule out neurological and developmental conditions. Informed consent was provided by the participants (adults), or a parent or legal guardian (children) verbally. In accordance with the Declaration of Helsinki, these experimental protocols were approved by the Johns Hopkins Institutional Review Board (IRB00276939).

Five hundred twenty-seven participants played Push ball, a task that allowed us to investigate the tradeoff between adaptation and online feedback control. Twenty-six participants did not finish the entire experiment and were removed from the final sample. Data sets were reviewed to ensure adequate sampling rates, data quality, and game play duration. Five participants were removed due to poor sampling rates on their home computers and poor data quality. Two additional participants were removed due to total game duration exceeding 2 h. This resulted in a final data set of 494 participants.

One hundred seventy-two additional participants, aged 5 and older, played Launch ball. We excluded 3-4yo participants from Launch ball because this age group had the most difficulty completing Push ball and were unlikely to be able to complete a more complicated task. Eight participants did not finish the entire experiment and were removed from the final sample. Two participants were removed due to poor sampling rates on their home computers and poor data quality. This resulted in a final data set of 162 participants.

The total sample analyzed came from 617 typically developing children and 39 young adults. Demographic information for participations is listed in Supplementary Table 1. Our sample includes participants from 42 states out of the 50 United States of America (Supplementary Fig. 12).

### Experimental protocol and data collection

Advances in remote data collection platforms have allowed for testing large cohorts of participants^68,72^. We utilized gamification techniques to design an experimental task that would engage children as young as three-years-old using short Disney cartoon video clips, and a naturalistic feel with colorful environment. Participants completed one of two tasks: Push ball or Launch ball. In Push ball, participants completed an error-based adaptation reaching task previously described and validated in Malone et al.^24^. Briefly, this game was developed using JavaScript to be run from a web browser to collect data remotely at home. Most children (61% who played Push ball and 54% who played Launch ball) used a mouse during the task. Supplementary Table 1 shows a breakdown of the percentage of children and adults who used a trackpad or touchscreen. We analyzed the effect of device, which is described in the Supplementary Results. The game was hosted on a customized website and data recording and storage was managed through Google Firebase Realtime Database.

We designed the video game task in a colorful, entertaining, 3D environment to make the experiment fun and engaging for children from toddler-age through adolescence. The games can be played online in a shortened version (https://kidmotorlearning.github.io/AdaptationTask_V8_Demo/index.html and https://kidmotorlearning.github.io/LaunchBall_V2_Demo/). All participants were provided with game instructions both in written and spoken formats within the game and were instructed to complete the task all the way through in a single session. Parents were instructed that they could provide motivation and encouragement to the children, but not to provide specific instructions on the task.

At the start of each trial, a still image of a different Disney cartoon video clip was presented on the screen; these were child-friendly gifs hosted on giphy.com. Participants used their dominant hand to move their finger or a computer mouse to control a ball (Fig. 1a) to contact the target (a play button) displayed centrally on the screen. Participants initiated a trial by activating the red game ball (located at 0,0 game units [GU] in the horizontal and vertical screen directions). The trial ended once the ball passed through the horizontal plane of the play button (24 GU). Note that ending within ±10 deg of the target center caused a short, child-friendly video clip to play (Fig. 1c, top). Missing the target caused a sad face and “Miss” to appear, and the video did not play (Fig. 1c, bottom). The distance of the movements with the mouse were dependent on the sensitivity and settings of the home computer and could not be recorded.

The tasks had four sequential blocks: baseline, learning, error clamp, and washout (Fig. 1b). During baseline and washout, participants received veridical feedback with no perturbation, meaning the ball position matched the participant’s movement. During learning, a 30-degree clockwise visuomotor rotation was gradually introduced over 60 trials (0.5 deg per trial) and then maintained for an additional 30 trials. This rotation created a mismatch between the participant’s movement and ball movement on the screen. Clockwise movements were defined as negative angles and counterclockwise positive angles. To be successful, participants had to learn to move the mouse to a location 30 degrees counterclockwise of the target so that the ball would move straight ahead and hit the target (see Fig. 1b, 2^nd^ panel in learning). During piloting, we tested out both gradual and abrupt rotations. Interestingly, with an abrupt rotation, we found that younger children would quit the game early due to frustration, thinking the game was broken. As a result, we proceeded with the gradual perturbation for the final experimental paradigm.

During the error clamp phase, the ball followed the y position of the mouse, but was displayed as moving nearly straight towards the target (with a small, random rotation of −2 to 2 degrees added each trial to mimic natural trial-to-trial variation). Therefore, each trial was guaranteed to be successful with little visuomotor error. This phase was used to assess retention of adaptation in absence of error signals^25,26^. Note that the veridical mouse movement was recorded during these trials for analysis. There was a brief break for participants between the baseline and learning block and between the error clamp and washout blocks. Participants transitioned seamlessly from the learning block into the error clamp block without any knowledge or instructions that the block had changed.

In Push ball, participants were able to use online visual feedback throughout their movement to make corrections in their trajectory *during the trial* since the ball followed the movement of the mouse throughout the trial (Fig. 1f). In contrast, for Launch ball participants made a movement with the mouse in the direction they wanted the ball to move, released the mouse click, and then the ball would launch in the direction of their movement along the Launch angle (Fig. 1d). This task was more akin to a “point and shoot” task. We recorded the entire movement trajectory from the participant, but the ball moved in a straight line along the Launch angle. In other words, for Push ball, participants were able to rely on the adaptation learning mechanism or online visual feedback to be successful in the task. In Launch ball, participants had to rely only on adaptation learning mechanisms. The comparison between these two tasks allowed us to investigate if any reliance on online feedback was a preference or more obligatory due to immature adaptation learning mechanisms in the children. Additionally, given that Launch ball was less naturalistic, participants received additional baseline trials to understand the game environment. After baseline, the two tasks were identical throughout the rest of the experiment.

Data sets were reviewed to ensure adequate sampling rates, data quality, and game play duration. Poor sampling rates on their home computers and poor data quality resulted in participants receiving inaccurate feedback of success or failure based due to low mouse polling or screen refresh rates. Game play duration was limited to less than 2 h as the task was intended to be completed a single time through without any prolonged breaks. Long breaks may result in forgetting or children doing other activities on their computer which would interfere with learning.

### Data analysis

We recorded the movement trajectory of the mouse (or trackpad, touchscreen) on every trial. The trajectory data sampled were in the form of change in (delta) mouse position based on the polling events of the device. When the mouse or finger was stationary, it would not poll and thus not provide data. The “movement angle” is defined as the angle between the (i) mouse to start location direction and (ii) the straight line from the start location to the target (defined as 0 degrees) (solid gray line, Fig. 1d).

We defined epochs throughout the experiment in order to quantify and compare behavior between the groups^73–78^. “Baseline” is the last 10 trials of the baseline block. “Learning” is the last 10 trials of the learning block. “Early-clamp” is the first 3 trials and “end-clamp” is the last 3 trials of the error clamp block respectively. “Early-washout” is the first 3 trials and “end-washout” is the last 10 trials of the washout block respectively. In order to adequately capture any transient differences between groups, we averaged fewer trials in epochs where we expected rapid changes in the behavior^38^.

Reaction time was defined as the time from trial onset to when the participant clicked the ball. Stationary time was the time from ball click until movement onset (Fig. 1f). Movement time was the time from movement onset to the end of the trajectory (final angle on Fig. 1f). We did not include stationary time as a part of movement time because children would sometimes click the ball and immediately start moving, whereas other times they would click the ball, wait a period of time, and then initiate movement.

For Push ball, on each trial, we derived a number of measures to characterize the movement. As has been done previously^27^^,79^, we used the initial angle (IA) to represent adaptation (Fig. 1d blue). The initial angle was calculated once participants moved the ball ≥1.2 GU (5%) towards the target. The final angle (the movement angle at the end of the trajectory, Fig. 1d purple) represented overall performance (including whether the trial was a hit or miss). The compensation angle (difference between the final and initial angle, Fig. 1d red) is typically considered a measure of the online feedback used during the movement to steer the ball^27^.

We calculated the path length ratio as a measure of path straightness, defined as the ball’s path length divided by the distance between the first and last point of the trajectory^30–33^. However, participants could achieve larger path length ratios in different ways. Children could have a lot of curvature to their movement, or they could have trajectories with curls and zigzags (Fig. 6a). In order to capture these behavioral differences, we defined correction parameters. The correction window was defined as the initial angle for the trial ±5 degrees (Fig. 6a, gray). The correction point was the first data sample whereby the trajectory fell outside this correction window (Fig. 6a, yellow). The normalized correction time parameter was the time to the correction point divided by the movement time. We normalized the time to correction because movement time varied across participants. In other words, this parameter was identifying how far into the movement participants started to correct. Angle spread is the difference between the maximum movement angle and the minimum movement angle in the trajectory from the initial angle to the final angle (Fig. 6a, brown).

For Launch ball, on each trial, we recorded the launch angle (Fig. 2D, blue). Because there was no online visual feedback, the size of the movements varied and were arbitrary in Launch ball. Only the angle of movement mattered. As a reminder, regardless of the curvature or trajectory drawn by the participant, the launch angle was calculated by taking the start and end positions of trajectory and calculating a straight-line movement angle. Similar to Push ball, angle spread is the difference between the maximum movement angle and the minimum movement angle in the trajectory from the initial angle to the final angle (Fig. 6a, brown).

### Statistical analysis

One-way and repeated measures ANOVAs were used to compare age binned data. If the assumption of sphericity was violated (Mauchly’s test *P* < 0.05), Greenhouse-Geisser corrections were applied. Post-hoc analyses were performed using Bonferroni test.

To model the average amount of learning and retention throughout childhood, both logarithmic $(f({ag}{e}_{{mo}})=a* \log ({ag}{e}_{{mo}})+b)$ and inverse $\left(f({ag}{e}_{{mo}})=a+\frac{b}{{ag}{e}_{{mo}}}\right)$ models were considered as nonlinear fits. The inverse model was a better fit for most of the parameters and is based upon the assumption that a plateau occurs in the data, which we felt was more biologically appropriate. An exponential $(f({ag}{e}_{{mo}})=a* \exp \left(\right.b* {ag}{e}_{{mo}})$ model was used to fit the standard deviation of the last 10 trials in baseline and learning and the compensation angle at the end of learning and early clamp. Adults were excluded from the fit analysis but are shown in figures for reference.

To assess the effect of age on the angle spread and normalized correction time, single and multiple linear regressions were performed. To analyze which parameters best predicted learning and retention of the initial and final angle, a stepwise linear regression was completed in SPSS. Candidate variables included age, sex, and baseline standard deviation of the initial angle and final angle.

SPSS (IBM) was used for all statistical analysis and the alpha level was set at *p* = 0.05. All data are reported as mean ± standard error unless otherwise specified. Standard error was chosen to represent the uncertainty of the true mean of each group and allows for visual comparison of differences or similarities between the groups.

### Percentile growth curves

Smooth sex-specific percentile curves were constructed for four measures of interest. The measures included the participant-specific mean and standard deviation of the initial angle at the end of learning and the participant-specific mean and the standard deviation of the final angle at the end of learning (i.e., calculated over the last 10 trials of the learning block). Generalized additive models for location, scale and shape (GAMLSS)^80^ were fitted in “gamlss” R package (https://cran.r-project.org/web/packages/gamlss/index.html). Specifically, Lambda-Mu-Sigma (LMS) method to estimate percentile cures curves were employed^81^. LMS method transforms data points for each age using a three-parameter transformation map under the assumption that each parameter depends smoothly on age. Then, the transformed data is modeled using a normal or t-distribution. The percentiles of the fitted distribution are mapped back onto the original scale. All analyses were performed using “lms” function in “gamlss”. The WHO has adopted the GAMLSS methodology for creating reference growth curves^82^. Diagnostic of the LMS fits have been evaluated using worm plots for normalized quantile residuals and z-scores of the first four central-moments calculated for each of nine data-driven age groups^83^ (Supplementary Fig. 11).

Percentile curves were constructed based on the best fit family through the “gamlss” function. “Gamlss” uses the Akaike information criterion (AIC) as a goodness-of-fit measure to find the best fit; lower values indicate a better fit. The combined male and female data sets for each measure of performance were used to pick the best fit family. Then that same family was used to generate the percentile curves for the male and female data sets individually. Candidate families include “NO” (denoting normal distribution) which models the mean and variance, the “BCCG” (Box-Cox Cole and Green) which models the mean, variance, and skewness, and the “BCT” (Box-Cox t) and “BCPE” (Box-Cox power exponential) which model the mean, variance, skewness, and kurtosis^84^. An important consideration to note is that if the response variable contains negative values and zeros then the function will use the default “NO” family.

During the exploration of the percentile curve models, 2 out of the 464 participants (10yo, 4yo) were determined to be outliers for the percentile curve generation and were excluded. First, the 10yo participants’ mean final angle was near 40 degrees and when including them in the percentile curves, the final angle had a non-physiologic “bump” (i.e., local increase and decrease in curves that was not transmitted throughout each curve). Additionally, for the final angle, there was one participant (3yo) who had a negative mean final angle value. As previously mentioned, with any negative response variable values, only the “NO” family could be used. This model only captures the mean and variance and does not include any skewness or kurtosis, which visually was present for the data. Generating the descriptive statistics for the distribution, the mean final angle at the end of learning, had a skewness statistic of −3.106 ± 0.11 (mean ± SE) and a kurtosis statistic of 13.49 ± 0.22. Values for skewness and kurtosis larger than 2 are considered to be significant and identify non-normal distributions^85^. Removing this one negative value allowed the function to fit the mean final angle data better with the “BCPE” family (AIC with “NO” family = 2492, AIC with “BCPE” family = 2269) (Supplementary Fig. 13). Thus, these 2 participants were excluded from all parameters for the percentile curve modeling.

## Supplementary information

Supplementary Material

## Data Availability

Data are available upon reasonable request to the corresponding author.

## Code availability

Matlab code is available upon reasonable request to the corresponding author.
