# Supplementary material for: The control of movement gradually transitions from feedback control to feedforward adaptation throughout childhood
Source: NPJ Sci Learn. 2025 Mar 11;10:13. doi: 10.1038/s41539-025-00304-7 (PMC11897242; doi:10.1038/s41539-025-00304-7)
Supplement: Supplementary file 1 — Supplementary Material [file 41539_2025_304_MOESM1_ESM.docx]

Supplementary Results:

*Push Ball:*

*Effects of sex, handedness, and device*

Some prior studies have reported sex-based differences on motor learning tasks^1,2^ whereas others have not^3^. In this study, we found that there were not any sex related differences in adaptation (i.e., initial angle). There was also no effect of handedness. We did find a significant effect of the device that participants used to complete the task; however, this effect was similar across age groups (i.e., no group x input device interaction). Those participants who completed the task with a touchscreen demonstrated smaller initial angles than those who completed the task with a mouse (p <0.001). Those participants that used a trackpad had the highest initial angles across the experiment (p <0.001). These effects on device are expected, since touchscreen participants could see the mismatch between their finger movement and the ball, likely lessening more implicit adaptation learning mechanisms a small amount^4,5^. See Supplementary table 3 for specific results of the initial angle.

For overall performance on the task (i.e., final angle), both males and females performed similarly. There was an effect of handedness on the final angle with left-handed individuals demonstrating slightly larger final angles when compared to right-handed individuals, although it is worth noting that there were only 32 participants that were left-handed compared to the 462 participants that were right-handed. The effect of handedness on the final angle was similar across the age groups. We also found an effect of device on the final angle. Similar to that in the initial angle, those that completed the task with a touchscreen demonstrated smaller final angles than those completing the task with a mouse (p = 0.03). Those participants that used a trackpad had the largest final angles across the experiment (p <0.001). Importantly, there was not an interaction effect between group x input device or epoch x group x device, confirming that the effect of device was similar across all age groups. Supplementary table 3 summarizes the statistical result for the final angle.

To further investigate the effects of touchscreen, we conducted a second analysis where the touchscreen participants were removed. After removing the touchscreen participants, we found that the results for the initial angle were largely the same as the entire cohort, except the early clamp 5-6yo group was not statistically different from the adults and the 3-4yo children did not quite reach significance (p=0.057) when compared to the adults at the end of clamp. For the initial angle, there was a significant effect for epoch (F_4.25,1816_ = 327.5, p < 0.001) and age-group (F_7,427_ = 3.76, p < 0.001), and interaction (F_29.8,1816_ = 3.16, p < 0.001). At the end of learning, post hoc analysis revealed that when compared to the adult group, the 3-4yo (p<0.001), 5-6yo (p<0.001), 7-8yo (p<0.001), 9-10yo (p=0.029), 11-12yo (p=0.027) all were statistically different, whereas the 13-14yo and 15-17yo groups were not. In early clamp, the initial angle was significantly different for the 3-4yo (p<0.001) but not the 5-6yo (p=0.18) when compared to the adults, indicating significantly less retention in the youngest age groups. The end of clamp between the 3-4yo and the adults did not reach significance (p = 0.057). Other epochs demonstrated no differences when compared to adults.

Similar to the initial angle, when removing the touchscreen participants, we found that the results for the final angle were the same except the early clamp 5-6yo group was not statistically different from the adults. For the final angle, there was a significant effect for epoch (F_2.86,1220_ = 1332, p < 0.001) and age-group (F_7,427_ = 5.97, p < 0.001), and interaction (F_20,1220_ = 2.98, p < 0.001). At the end of learning, post hoc analysis revealed that the 3-4yo (p<0.001) were the only group that was different than adults. In early clamp, the final angle was significantly different for the 3-4yo (p<0.001) but not the 5-6yo (p=0.19) when compared to the adults. The end of clamp between the 3-4yo and the adults was also significant (p = 0.03). Other epochs demonstrated no differences when compared to adults.

For the compensation angle, results were very similar even when removing the touchscreen participants with only the 9-10yo group being close to significance (p=0.055 versus p=0.007). For the compensation angle, there was a significant effect for epoch (F_4.5,1920_ = 90.48, p < 0.001) and an epoch x group interaction (F_31.5,1920_ = 1.77, p =0.005), but not for age-group (F_7,427_ = 0.66, p = 0.71) alone. At the end of learning, post hoc analysis revealed that when compared to the adult group, the 3-4yo (p<0.001), 5-6yo (p=0.007), 7-8yo (p=0.007), 11-12yo (p=0.03) all were statistically different, with the 9-10yo being close to significance (p=0.055). The 13-14yo and 15-17yo groups were not different than adults.

*Timing parameters*

Studies of motor learning commonly report timing parameters of the movement in addition to spatial parameters. We chose to use spatial parameters as our primary measures due to irregular sampling rates from home computers^6^; however, here we also report on timing parameters to further describe participant behavior. Reaction time was defined as the time from trial onset to when the participant clicked the ball. Stationary time was the time from ball click until movement onset (Figure 1f). Movement time was the time from movement onset to the end of the trajectory (final angle on Figure 2f Push ball). We did not include stationary time as a part of movement time because children would sometimes click the ball and immediately start moving, whereas other times they would click the ball, wait a period of time, and then initiate movement. With regards to timing parameters, we found that younger children had longer reaction times (F_7,486_ = 27.4, p<0.001) and movement times (F_7,486_ = 15.9, p<0.001) when compared to older children and adults (Supplementary Figure 2). There was also a significant effect of group on stationary time (F_7,486_ = 2.96, p=0.005); however, post hoc testing only showed a difference between the 3-4yo group and the 15-17yo group that did not quite reach significance (p =0.056, all other p’s >0.1). While there was a significant effect of age-group on the IA_time_ (F_7,486_ = 7.33, p<0.001), the time to initial angle calculation was longer in younger children when compared to older age groups. On average, the IA_time_ was 260ms across all groups. Despite the initial angle being calculated later in the movement for younger children (e.g., IA_time_ of 3-4yo: 445 ± 51ms), we still find that younger children adapted less. If the initial angle calculation in the younger groups did include some online feedback corrections, their true adaptation ability would be even less than what is observed here.

*Predictors of learning and performance*

To investigate the influence of age, sex, and baseline variability on learning, we completed stepwise linear regressions. Dependent variables were adaptation and overall performance (i.e., the mean of the initial angle and the mean of the final angle at the end of learning). Possible predictors included age in months, sex, baseline standard deviation of the initial angle, and baseline standard deviation of the final angle. For adaptation, only age and the baseline standard deviation of the final angle were included in the best fit model (F_2,491_ = 46.6, p<0.001, adjusted r^2^ = 0.16). Age was the best predictor with an r^2^ change of 0.15; baseline standard deviation of the final angle r^2^ change was only 0.009. Standard deviation of the initial angle at baseline and sex were excluded. Thus, the main predictors of adaptation were age and the variability of overall performance at baseline (indicative of general motor control).

For overall performance (includes adaptation and online corrections), baseline standard deviation of the final angle, age, and sex were included in the best fit model (F_3,490_ = 47.5, p<0.001, adjusted r^2^ = 0.22). Standard deviation of the initial angle at baseline did not reach significance in the model. Thus, good overall performance was predicted by baseline precision and males of older age. Baseline standard deviation of the final angle was the best predictor with an r^2^ change of 0.19, with age next with an r^2^ change of 0.03, and sex with an r^2^ change of 0.007. In contrast to adaptation, the overall performance at the end of learning was influenced mostly by variability of overall performance at baseline, with much smaller effects of age and sex.

In the clamp phase we saw that the initial and final angles were similar (Supplementary Figure 9a and 9b), both reflecting the retention of adaptation. If instead, we use data from *the early clamp* epoch, we find that age is again the best predictor of retention for both initial (F_1,492_ = 35.1, p <0.001, r^2^ change = 0.07) and final angles (F_2,491_ = 33.9, p <0.001, r^2^ change = 0.09). Recall that in the clamp phase both the initial and final angle could be used to measure retention of the adaptation learning mechanism. No other predictors were included in the best fit model for the initial angle, but the final angle did include the standard deviation of the final angle at baseline (r^2^ change = 0.03). Thus, the final angle during early clamp and the initial angle at the end of learning have similar models. This analysis confirms that either the initial angle at the end of learning or the initial or final angle at the early clamp could be used to measure adaptation. Previously, we discussed that we chose to use the end of learning as our primary outcome measure instead of the clamp trials because behavior was more stable.

Launch ball:

*Age group effects across epochs*

Note that the sample size is significantly smaller for Launch ball, and thus we have less power to investigate age group related effects. For the launch angle, there was a significant effect for epoch (F_3.03,469.3_ = 144.97, p < 0.001) but not age-group (F_6,155_ = 1.47, p < 0.19), and the interaction did not quite reach significance (F_18.2,469.3_ = 4.54, p < 0.08) (Supplementary Figure 5). There were no differences in baseline across age-groups, indicating all children and adults started with similar behavior in Launch ball. When investigating individual age group post-hoc effects, all groups demonstrated significant effects between baseline and the end of learning (all p’s<0.001).

To confirm the presence of after-effects within each age group, we investigated post-hoc effects of the launch angle between the clamp and washout blocks when compared to baseline. Collapsing across all groups, we found significant differences between early clamp and baseline (p<0.001), end clamp and baseline (p<0.001), early washout and baseline (p<0.001), and even the end washout and baseline (p<0.001). All groups demonstrated significant differences between early clamp and baseline (all p’s <0.001), indicating after-effects of the sensorimotor recalibration during Launch ball. All the children demonstrated significant differences between the end of clamp and baseline (all p’s ≤0.006) with the adult group nearing significance (p=0.059). Younger age groups demonstrated that this after-effect persisted longer throughout the washout period. In early washout, all the children demonstrated persistent after-effects (5-6yo, p=0.002; 7-8yo, p<0.001; 9-10yo, p=0.005; 11-12yo, p<0.001; 13-14yo, p=0.04; 15-17yo, p=0.045) while the adult group did not (p=0.19). In late washout, only the youngest groups still had differences in their launch angle when compared to baseline (5-6yo: p<0.001, 7-8yo: p=0.002) whereas older school age children through adulthood returned to baseline performance (all p’s >0.01). Similar to the findings in Push ball, we found that when compared to baseline, all age groups demonstrated after-effects in the clamp and washout blocks, with the older children and adults returning back to baseline performance by the end of washout while the younger children did not.


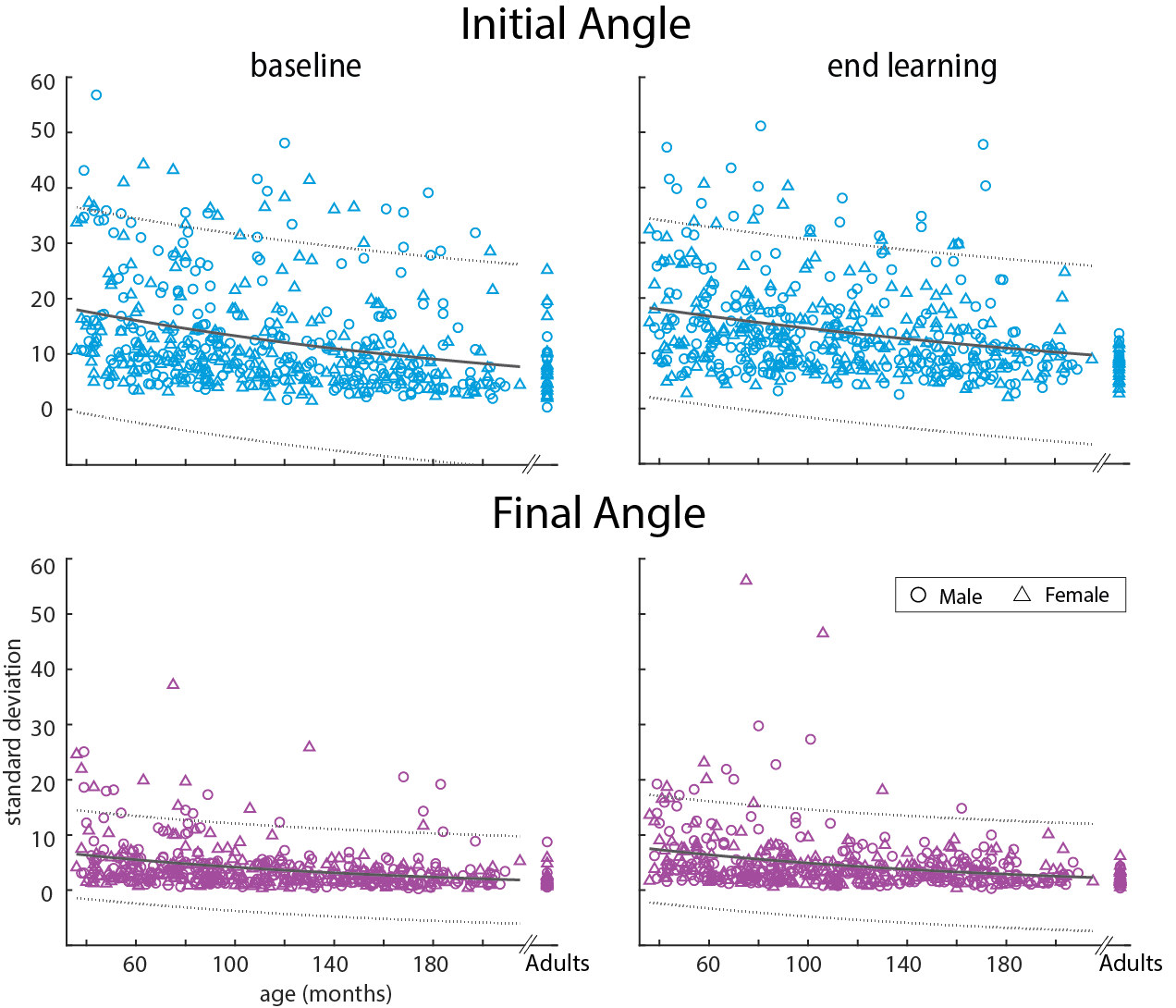


Supplementary Figure 1. Standard deviation (i.e., a measure of precision) at the end of learning and at baseline of the initial and final angle in Push ball with exponential model fits. Individual participant data are show for males (circles) and females (triangles) by age with adults shown on the right-hand side of the plot. Note that the adults were not included in the fits. Inverse model fits (solid gray line) and the 95% confidence intervals (dotted gray lines). For the initial angle at baseline, the model fit the parameters: a = 21.35 (17.97, 24.72), b = -0.0047 (-0.0063, -0.0032) with an adjusted R^2^ = 0.08. The final angle at the end of baseline the model fit the parameters: a = 8.41 (6.64, 10.19), b = -0.0070 (-0.0093, -0.0048) with an adjusted R^2^ = 0.08. For the end of learning, the initial angle fit was a = 20.67 (18, 23.33), b = -0.0035 (-0.0048, -0.0023) with an adjusted R^2^ = 0.07 and the final angle fit was a = 9.54 (7.44, 11.63), b = -0.0066 (-0.0089, -0.0043) with an adjusted R^2^ = 0.07.


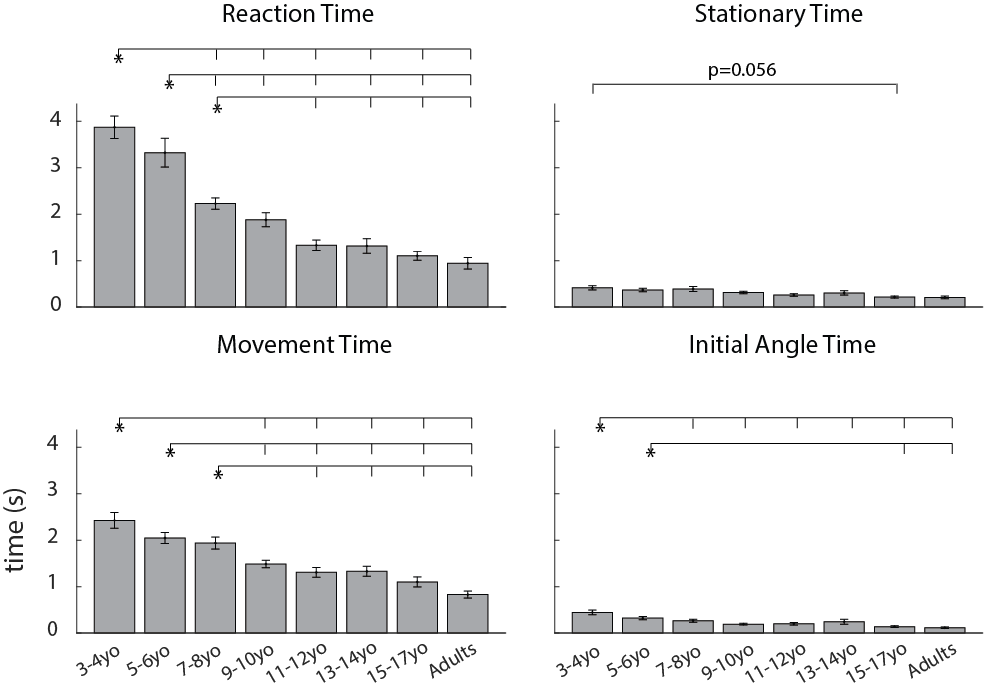
Supplementary Figure 2. Push ball timing parameters. Timing parameters (mean ± s.e. across the participants, data was first averaged for all trials within each participant) comparing age bins for Push ball. Statistically significant differences are shown by an asterisk (p<0.05).


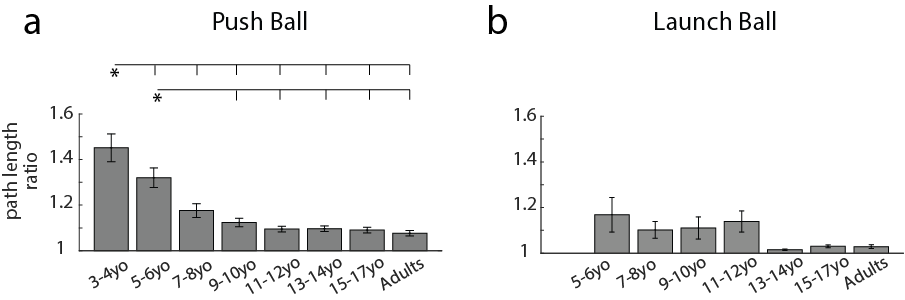


Supplementary Figure 3. Path length ratio (mean ± s.e) over all trials for Push ball (a) and Launch ball (b). Path length ratio was defined as the path length divided by the distance between the first and last point of the path. Statistically significant differences are shown by an asterisk (p<0.05).


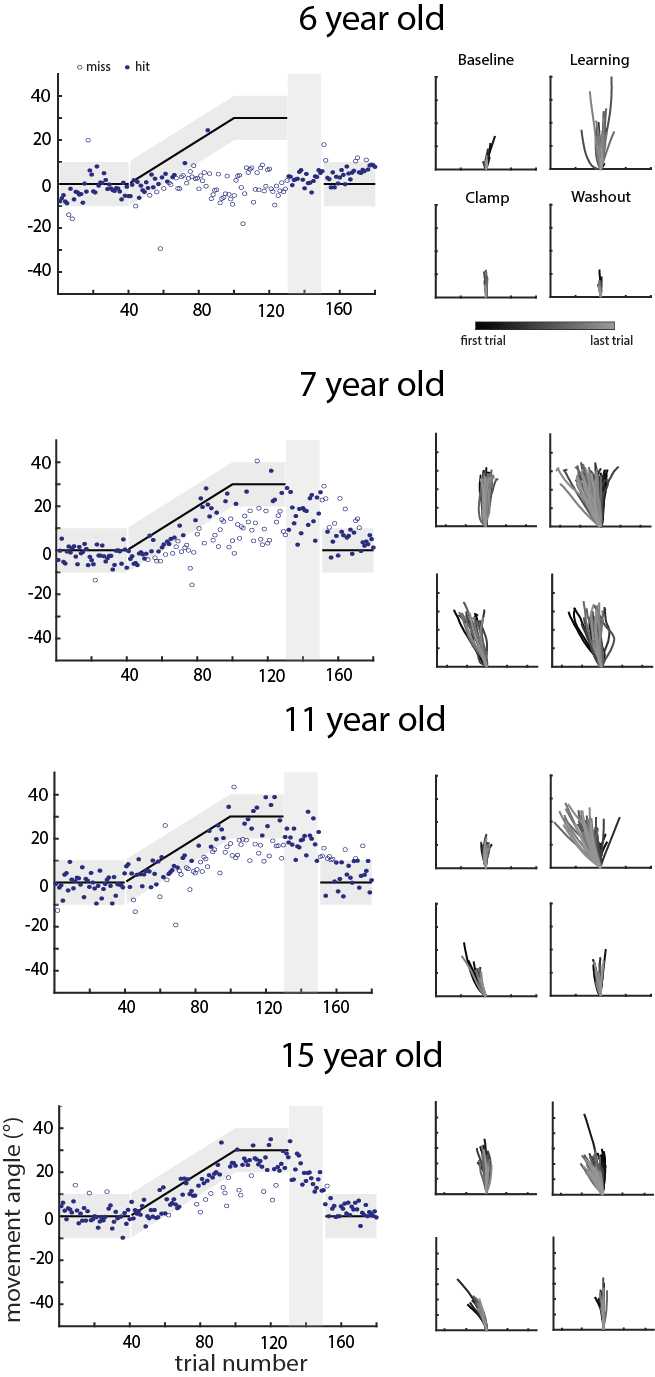


Supplementary Figure 4. Example Launch ball trial-by-trial data and trajectories of individual participants. Filled circles are successful trials and open circles are unsuccessful trials. Trajectory data is displayed for each block and earlier trials are darker than later trials. Note that most of the trajectories are straight as opposed to curved, even during learning.


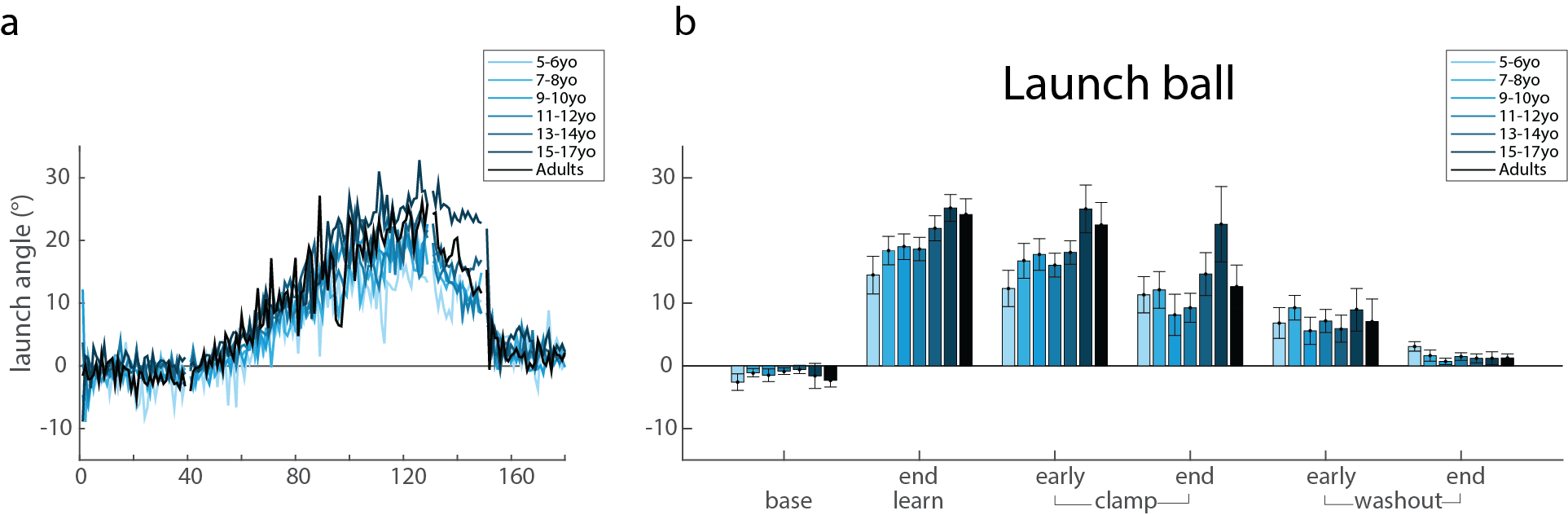


Supplementary Figure 5. Launch ball overlaid trial-by-trial group averages and experimental epochs by age bin. Trial-by-trial group averages (a) and epochs (b) of the launch angle by age bin throughout the experimental paradigm. Mean ± s.e. are shown in the epoch bar plots. Note that there is no 3-4yo age group for Launch ball. Younger children are lighter colors compared to adolescents in darker colors and adults are in black. Even in Launch ball, we found that all participant age groups demonstrated after-effects in the early clamp compared to baseline.


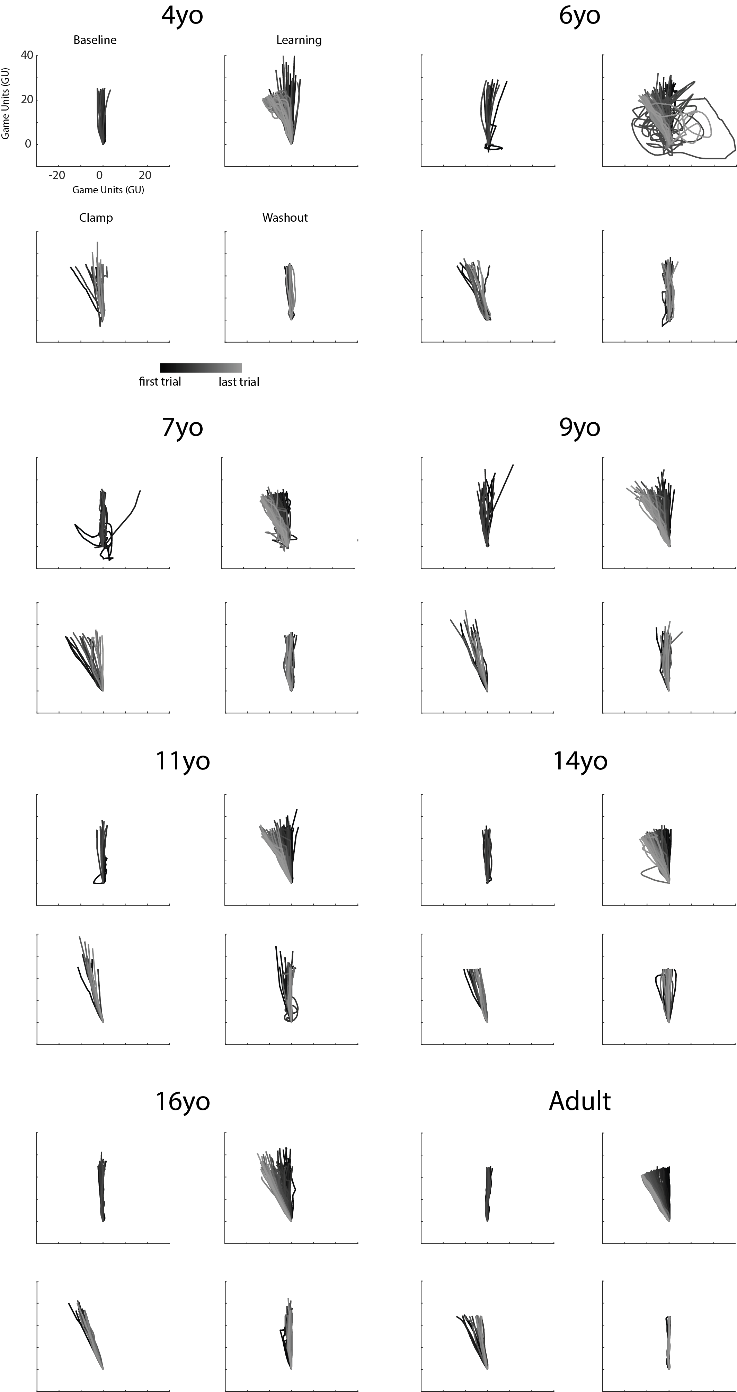


Supplementary Figure 6. Push ball trajectories of example participants from each age bin throughout the experiment (baseline, learning, clamp, and washout). Darker colors represent earlier trials; lighter colors are at the end of the block.


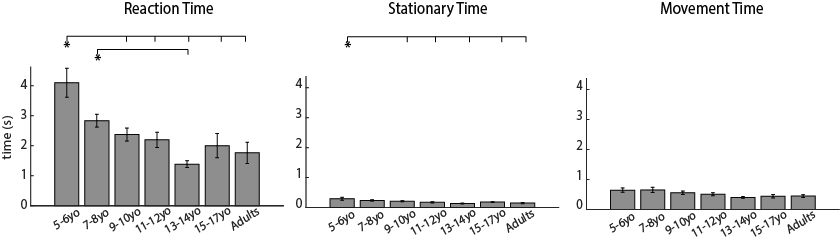


Supplementary Figure 7. Timing parameters for Launch ball. Timing parameters (mean ± s.e. across the participants, data was first averaged for all trials within each participant) comparing age bins for Launch ball. Statistically significant differences are shown by an asterisk (p<0.05).


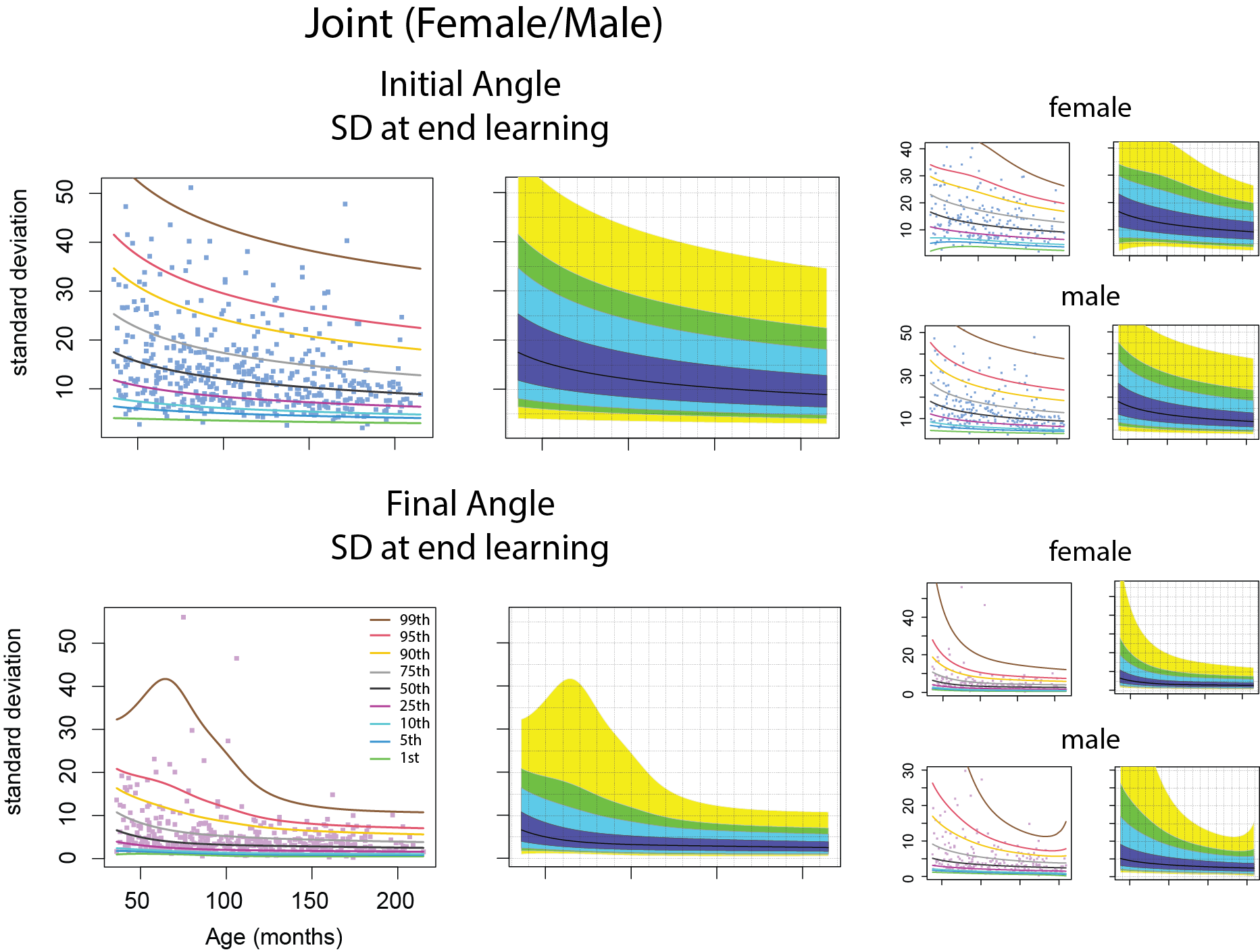


Supplementary Figure 8. Percentile curve estimation for standard deviation of the initial and final angle at the end of learning of all children and separated by female and male from Push ball data. Adults are not included. Data from individual children are shown in the filled boxes with the percentile estimates shown in the solid lines. The percentile curve estimates are also demonstrated in the fan plot without data shown to better show the pattern of age-based effects.


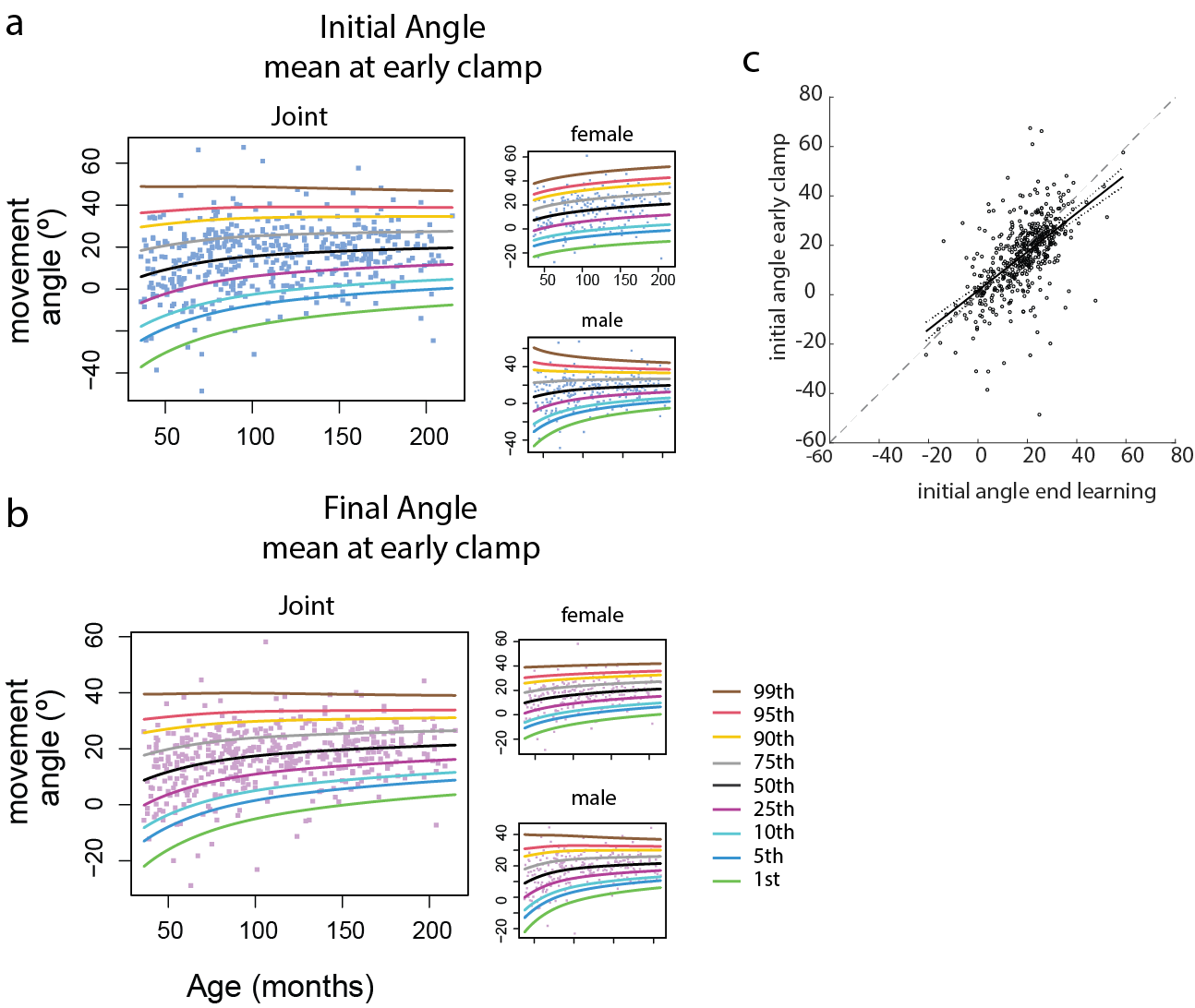


Supplementary Figure 9. Percentile curve estimation for mean of the initial (a) and final angle (b) in the early clamp of all children and separated by female and male from Push ball data. Adults are not included. Data from individual children are shown in the filled boxes with the percentile estimates shown in the solid lines. The initial and final angle look very similar in the early clamp epoch. c) Relationship between the initial angle at the end of learning compared to the initial angle in early clamp. Linear model fit (solid black line) and 95% confident intervals (dotted black lines) are shown in addition to the unity line (dotted gray line). The initial angle at the end of learning has a smaller spread, reflecting more stability in the parameter. There is a significant linear correlation between the initial angle at end of learning and early clamp (y=0.79x + 1.58; F_1,492_ = 282.5, p <0.001, adjusted r^2^ = 0.36).


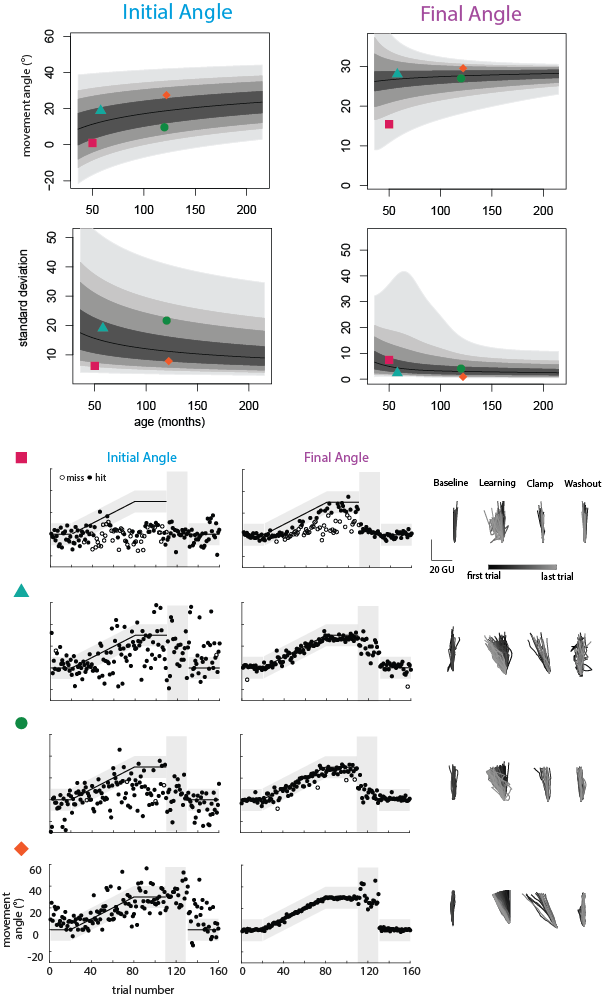


Supplementary Figure 10. Examples of individual participant performance plotted on gray-scale fan percentile curves for the initial and final angle at the end of learning with their respective trial-by-trial and trajectory performance. Two 4-year-old (pink square and blue triangle) and two 10-year-old (green circle and orange diamond) participants are shown.


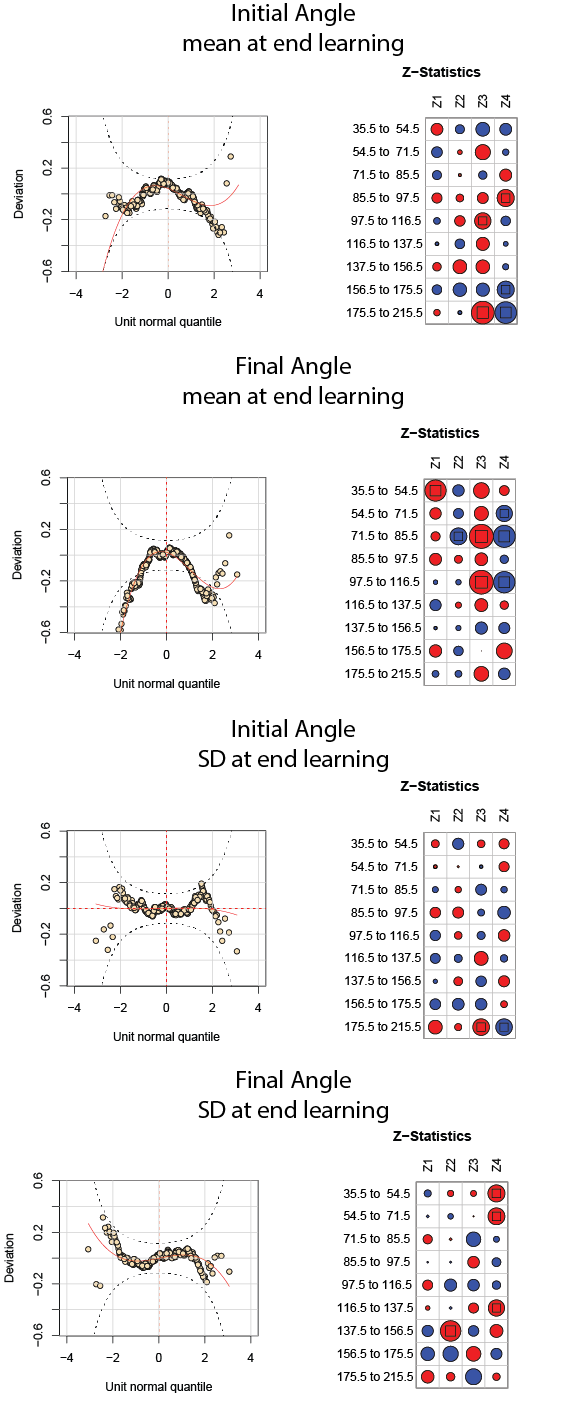


Supplementary Figure 11. Worm plots and Z-statistics for percentile curves. Worm plots (left) and Z-statistics (right) for joint female and male percentile curve fits for both mean and standard deviation of the initial and final angle at the end of learning from Push ball data. For the worm plots, the more data that stays in the center of the plot (around 0 deviation) and outside of the two dotted half-circles, the better the fit of the model. Smaller size of the circle indicates a smaller z-statistics and smaller deviation from the underlying assumptions. Circles with a box inside are the largest residuals (>1.96). Red is negative residuals and blue is positive.


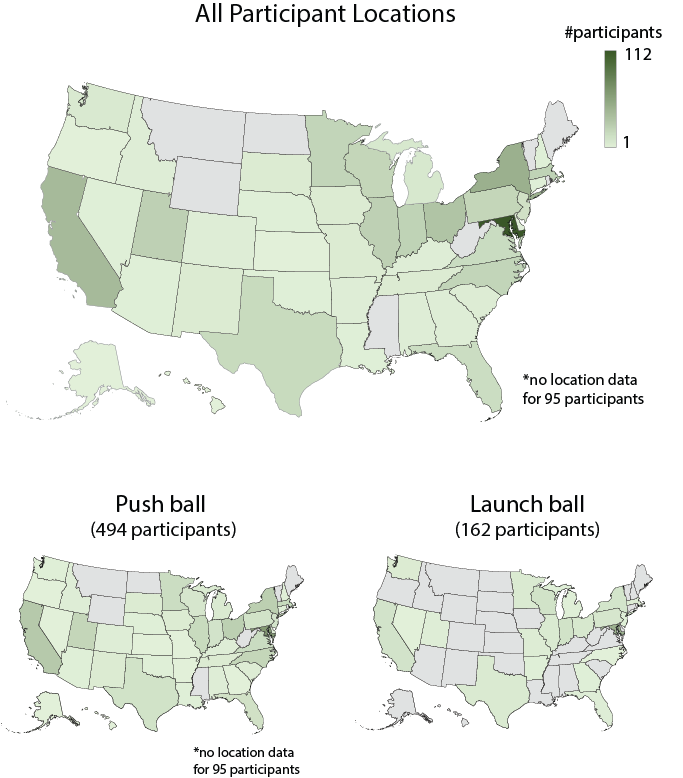


Supplementary Figure 12. Map of participant locations that participated in the experiment. Darker green indicates more participants in the state.


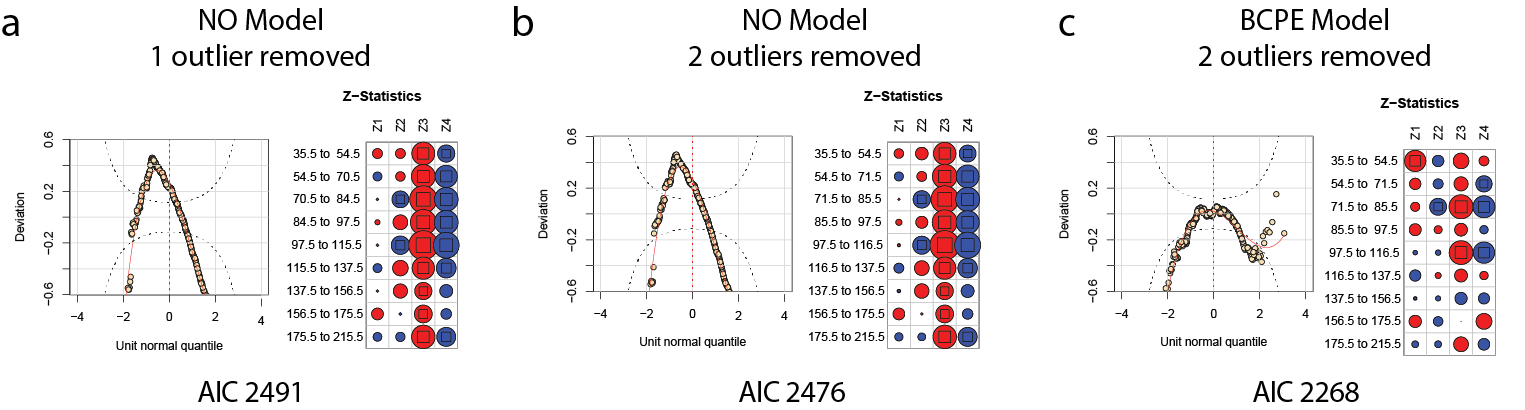


Supplementary Figure 13. Worm plots and Z-statistics for NO model with single outlier removed (a), both outliers removed (b) compared with the BCPE Model with both outliers removed (c) for the mean final angle at the end of learning. Circles with a box inside are the largest residuals (>1.96). Red is negative residuals and blue is positive. By excluding the one participant with a negative final angle at the end of learning, we were able to obtain a much better fit to the data.

| Task | Push ball | | | Launch ball | |
| --- | --- | --- | --- | --- | --- |
|  | Kids | Adults | | Kids | Adults |
| n | 464 | | 30 | 153 | 9 |
| % Female | 45.3 | | 66.7 | 43.8 | 66.7 |
| % Right-Handed | 93.1 | | 100 | 94.9 | 100 |
|  |  | |  |  |  |
| **Age (yrs)** |  | |  |  |  |
| Mean (std) | 14.9 (3.9) | | 26.1 (4.2) | 10.4 (2.9) | 23.7 (4.3) |
| Median | 9 | | 25.2 | 10.5 | 23.5 |
| Range | 3.0-17.9 | | 18.7 -34.8 | 5.0-17.6 | 18.8-30.4 |
|  |  | |  |  |  |
| **N per age bin** |  | |  |  |  |
| 3-4yo | 71 | |  | 0 |  |
| 5-6yo | 79 | |  | 21 |  |
| 7-8yo | 79 | |  | 33 |  |
| 9-10yo | 73 | |  | 33 |  |
| 11-12yo | 56 | |  | 35 |  |
| 13-14yo | 64 | |  | 21 |  |
| 15-17yo | 42 | |  | 10 |  |
| 18yo+ |  | | 30 |  | 9 |
|  |  | |  |  |  |
| **Device (%)** |  | |  |  |  |
| Mouse | 60.6 | | 76.7 | 53.6 | 88.9 |
| Trackpad | 26.7 | | 23.3 | 32.7 | 11.1 |
| Touchscreen | 12.7 | | 0 | 13.7 | 0 |
|  |  | |  |  |  |
| **Race (%)** |  | |  |  |  |
| Black | 4.3 | | | 1.2 | |
| White | 75.1 | | | 65.4 | |
| Asian | 7.9 | | | 16 | |
| Multiple | 7.1 | | | 8 | |
| Other | 5.6 | | | 9.3 | |
|  |  | |  |  |  |
| **Ethnicity (%)** |  | |  |  |  |
| Hispanic | 5.9 | | | 6.2 | |
| Non-Hispanic | 94.1 | | | 93.8 | |

Supplementary Table 1. Participant characteristics and demographics for Push ball and Launch ball.

| Age (mo) | Percentile | | | | | | | | |
| --- | --- | --- | --- | --- | --- | --- | --- | --- | --- |
|  | 1 | 5 | 10 | 25 | 50 | 75 | 90 | 95 | 99 |
| Mean Initial Angle at the End of Learning | | | | | | | | | |
| 36 | -21.533973 | -12.710775 | -8.00715998 | -0.1476262 | 8.584886 | 17.3174 | 25.17693 | 29.88055 | 38.70375 |
| 48 | -17.332512 | -9.0335123 | -4.60934635 | 2.783241 | 10.996942 | 19.21064 | 26.60323 | 31.0274 | 39.3264 |
| 60 | -14.147282 | -6.2332914 | -2.01437228 | 5.0352566 | 12.867906 | 20.70056 | 27.75018 | 31.9691 | 39.88309 |
| 72 | -11.590378 | -3.9775936 | 0.08075362 | 6.8620738 | 14.396613 | 21.93115 | 28.71247 | 32.77082 | 40.3836 |
| 84 | -9.4593668 | -2.0922164 | 1.83518418 | 8.3976984 | 15.689128 | 22.98056 | 29.54307 | 33.47047 | 40.83762 |
| 96 | -7.6355087 | -0.4746571 | 3.34276636 | 9.7215136 | 16.808765 | 23.89602 | 30.27476 | 34.09219 | 41.25304 |
| 108 | -6.0433049 | 0.9404295 | 4.66343245 | 10.8844072 | 17.796362 | 24.70832 | 30.92929 | 34.65229 | 41.63603 |
| 120 | -4.6318292 | 2.1972196 | 5.83776023 | 11.920944 | 18.679803 | 25.43866 | 31.52185 | 35.16239 | 41.99143 |
| 132 | -3.3651459 | 3.326948 | 6.89447851 | 12.8556655 | 19.478977 | 26.10229 | 32.06348 | 35.63101 | 42.3231 |
| 144 | -2.2169823 | 4.3524929 | 7.85465587 | 13.7066166 | 20.20857 | 26.71052 | 32.56248 | 36.06465 | 42.63412 |
| 156 | -1.1675679 | 5.2911002 | 8.73419235 | 14.4874483 | 20.879733 | 27.27202 | 33.02527 | 36.46837 | 42.92703 |
| 168 | -0.2016577 | 6.1560885 | 9.54537947 | 15.2087361 | 21.501136 | 27.79354 | 33.45689 | 36.84618 | 43.20393 |
| 180 | 0.6927443 | 6.9579526 | 10.29791201 | 15.8788377 | 22.079651 | 28.28046 | 33.86139 | 37.20135 | 43.46656 |
| 192 | 1.525241 | 7.7051053 | 10.99956815 | 16.5044711 | 22.620818 | 28.73716 | 34.24207 | 37.53653 | 43.71639 |
| 204 | 2.3036388 | 8.4043936 | 11.65668346 | 17.0911172 | 23.129168 | 29.16722 | 34.60165 | 37.85394 | 43.9547 |
| 216 | 3.0349926 | 9.0620174 | 12.27500204 | 17.6437585 | 23.608837 | 29.57391 | 34.94267 | 38.15566 | 44.18268 |
| Mean Final Angle at the End of Learning | | | | | | | | | |
| 36 | 9.012688 | 16.5996 | 19.87961 | 23.84448 | 26.22619 | 28.69221 | 33.17545 | 37.32098 | 49.3156 |
| 48 | 10.437404 | 17.73989 | 20.76638 | 24.33173 | 26.57732 | 28.76548 | 31.82728 | 34.15865 | 39.49056 |
| 60 | 12.466449 | 18.94749 | 21.60638 | 24.76002 | 26.85286 | 28.80362 | 31.13167 | 32.73691 | 36.07132 |
| 72 | 14.067333 | 19.91063 | 22.27715 | 25.10774 | 27.0692 | 28.82776 | 30.7095 | 31.9282 | 34.3241 |
| 84 | 15.342904 | 20.68864 | 22.82351 | 25.39676 | 27.24451 | 28.84868 | 30.43138 | 31.41156 | 33.26826 |
| 96 | 16.404038 | 21.34036 | 23.28414 | 25.64458 | 27.39183 | 28.87053 | 30.23924 | 31.05815 | 32.56694 |
| 108 | 17.322399 | 21.90387 | 23.68386 | 25.86242 | 27.51932 | 28.89422 | 30.10231 | 30.80509 | 32.07168 |
| 120 | 18.143037 | 22.403 | 24.03828 | 26.05741 | 27.63203 | 28.91948 | 30.00248 | 30.61775 | 31.70645 |
| 132 | 18.894713 | 22.85296 | 24.35758 | 26.23438 | 27.73339 | 28.94586 | 29.92862 | 30.47557 | 31.42829 |
| 144 | 19.595713 | 23.26361 | 24.64859 | 26.39673 | 27.8258 | 28.97299 | 29.8735 | 30.36567 | 31.21119 |
| 156 | 20.257158 | 23.64149 | 24.91609 | 26.54698 | 27.9111 | 29.00068 | 29.83241 | 30.27965 | 31.03857 |
| 168 | 20.884877 | 23.99097 | 25.16347 | 26.68705 | 27.99069 | 29.02885 | 29.80215 | 30.21188 | 30.89943 |
| 180 | 21.480662 | 24.31492 | 25.39314 | 26.81832 | 28.06553 | 29.05738 | 29.78036 | 30.1583 | 30.78606 |
| 192 | 22.043505 | 24.61532 | 25.60682 | 26.94175 | 28.1363 | 29.08609 | 29.76525 | 30.11591 | 30.69288 |
| 204 | 22.570927 | 24.89367 | 25.80585 | 27.0581 | 28.20348 | 29.11486 | 29.75548 | 30.08246 | 30.61578 |
| 216 | 23.060973 | 25.15166 | 25.99162 | 27.16811 | 28.2675 | 29.14355 | 29.74996 | 30.05617 | 30.55155 |
| SD Initial Angle at the End of Learning | | | | | | | | | |
| 36 | 4.025704 | 6.424208 | 8.131269 | 11.83146 | 17.51032 | 25.32508 | 34.68635 | 41.5679 | 57.59146 |
| 48 | 3.92123 | 6.03323 | 7.530855 | 10.78333 | 15.81771 | 22.85042 | 31.43546 | 37.85175 | 53.10722 |
| 60 | 3.81707 | 5.727116 | 7.078027 | 10.01594 | 14.59246 | 21.0605 | 29.07654 | 35.14945 | 49.84489 |
| 72 | 3.719559 | 5.476906 | 6.717371 | 9.41796 | 13.64612 | 19.67897 | 27.25106 | 33.05459 | 47.31604 |
| 84 | 3.629756 | 5.266166 | 6.419452 | 8.932388 | 12.88322 | 18.56591 | 25.77704 | 31.36053 | 45.27191 |
| 96 | 3.547293 | 5.08471 | 6.166824 | 8.52635 | 12.24917 | 17.64134 | 24.55026 | 29.94877 | 43.56959 |
| 108 | 3.471444 | 4.925801 | 5.948327 | 8.179267 | 11.71004 | 16.85557 | 23.50583 | 28.74546 | 42.11994 |
| 120 | 3.401448 | 4.78476 | 5.756413 | 7.87746 | 11.24342 | 16.17577 | 22.60083 | 27.7017 | 40.86382 |
| 132 | 3.336613 | 4.658216 | 5.58575 | 7.611413 | 10.83377 | 15.57922 | 21.80551 | 26.78355 | 39.76019 |
| 144 | 3.276334 | 4.54366 | 5.432442 | 7.374256 | 10.46996 | 15.04961 | 21.09848 | 25.9666 | 38.77948 |
| 156 | 3.220089 | 4.439172 | 5.293551 | 7.160878 | 10.14373 | 14.57484 | 20.46388 | 25.23271 | 37.89973 |
| 168 | 3.16743 | 4.343251 | 5.166813 | 6.967374 | 9.84878 | 14.14574 | 19.88964 | 24.56812 | 37.10422 |
| 180 | 3.117973 | 4.254703 | 5.050446 | 6.790702 | 9.58025 | 13.75518 | 19.36639 | 23.96209 | 36.37998 |
| 192 | 3.071386 | 4.172561 | 4.943023 | 6.628448 | 9.334279 | 13.39752 | 18.88672 | 23.40616 | 35.71672 |
| 204 | 3.027385 | 4.096031 | 4.843385 | 6.478664 | 9.107764 | 13.06823 | 18.44467 | 22.89349 | 35.10619 |
| 216 | 2.985666 | 4.024373 | 4.750471 | 6.339608 | 8.897959 | 12.76331 | 18.03495 | 22.41801 | 34.54093 |
| SD Final Angle at the End of Learning | | | | | | | | | |
| 36 | 0.9625475 | 1.7669728 | 2.404181 | 3.933526 | 6.599164 | 10.766475 | 16.370846 | 20.849767 | 32.27571 |
| 48 | 1.1342921 | 1.7100339 | 2.150517 | 3.210567 | 5.158668 | 8.582739 | 14.045097 | 19.195741 | 35.93306 |
| 60 | 1.1025082 | 1.5589319 | 1.903531 | 2.732487 | 4.290388 | 7.205249 | 12.407603 | 17.984491 | 41.06369 |
| 72 | 1.0059672 | 1.4054787 | 1.704794 | 2.421206 | 3.766311 | 6.308737 | 10.973618 | 16.169268 | 39.70064 |
| 84 | 0.8675813 | 1.2536248 | 1.539705 | 2.214731 | 3.453128 | 5.722732 | 9.731924 | 14.038994 | 32.31244 |
| 96 | 0.7198798 | 1.1141581 | 1.402998 | 2.071212 | 3.255937 | 5.331617 | 8.810968 | 12.37682 | 26.3657 |
| 108 | 0.6178187 | 1.0150376 | 1.306327 | 1.972931 | 3.121103 | 5.034142 | 8.019414 | 10.86378 | 20.68307 |
| 120 | 0.5685728 | 0.9585246 | 1.247251 | 1.9074 | 3.023323 | 4.796859 | 7.355384 | 9.595015 | 16.28799 |
| 132 | 0.5390192 | 0.9214661 | 1.206684 | 1.858575 | 2.945907 | 4.616715 | 6.898688 | 8.784735 | 13.93125 |
| 144 | 0.5194821 | 0.8951207 | 1.176143 | 1.817531 | 2.877423 | 4.472702 | 6.58364 | 8.273274 | 12.6671 |
| 156 | 0.5091605 | 0.8769424 | 1.152201 | 1.779512 | 2.810638 | 4.34595 | 6.345161 | 7.919818 | 11.91864 |
| 168 | 0.507551 | 0.8655998 | 1.133247 | 1.742723 | 2.743026 | 4.227835 | 6.151283 | 7.657729 | 11.44946 |
| 180 | 0.5127733 | 0.8596239 | 1.118356 | 1.707515 | 2.676022 | 4.11745 | 5.989494 | 7.457966 | 11.15773 |
| 192 | 0.5210931 | 0.8564573 | 1.106021 | 1.674535 | 2.612154 | 4.015759 | 5.851436 | 7.299676 | 10.97338 |
| 204 | 0.5294685 | 0.8539347 | 1.094821 | 1.64384 | 2.552645 | 3.922467 | 5.729551 | 7.165984 | 10.84444 |
| 216 | 0.5365358 | 0.8510267 | 1.083999 | 1.6152 | 2.497568 | 3.836545 | 5.61864 | 7.046457 | 10.74079 |

Supplementary Table 2. Table of percentile estimates by age in months for the mean and SD of the initial and final angles at the end of learning.

| Initial Angle | | | |
| --- | --- | --- | --- |
| Sex | Sex | F_1,478_ = 0.4 | p = 0.53 |
|  | Group x sex | F_7,478_ = 0.42 | p = 0.89 |
|  | Epoch x sex | F_4.23,2019_ = 0.4 | p = 0.82 |
|  | Epoch x group x sex | F_29.6,2019_ = 0.73 | p =0.86 |
| Handedness | Handedness | F_1,480_ = 0.66 | p = 0.42 |
|  | Group x handedness | F_5,480_ = 1.1 | p = 0.35 |
|  | Epoch x handedness | F_4.22,2025.7_ = 0.882 | p = 0.48 |
|  | Epoch x group x handedness | F_21.1,2025.7_ = 0.63 | p = 0.90 |
| Device | Device | F_2,471_ = 42.1 | p <0.001 * |
|  | Group x device | F_13,471_ = 0.81 | p = 0.651 |
|  | Epoch x device | F_8.6,2022.7_ = 8.4 | p <0.001 * |
|  | Epoch x group x device | F_55.8,2022.7_ = 0.71 | p = 0.95 |

| Final Angle | | | |
| --- | --- | --- | --- |
| Sex | Sex | F_1,478_ = 1.6 | p = 0.20 |
|  | Group x sex | F_7,478_ = 0.43 | p = 0.88 |
|  | Epoch x sex | F_2.86,1365.7_ = 0.34 | p = 0.79 |
|  | Epoch x group x sex | F_20,1365.7_ = 0.90 | p =0.59 |
| Handedness | Handedness | F_1,480_ = 9.3 | p = 0.002 * |
|  | Group x handedness | F_5,480_ = 0.49 | p = 0.79 |
|  | Epoch x handedness | F_2.9,1380.1_= 2.66 | p = 0.049 * |
|  | Epoch x group x handedness | F_14.4,1380.1_ = 0.43 | p = 0.97 |
| Device | Device | F_2,471_ = 15 | p <0.001 * |
|  | Group x device | F_13,471_ = 0.58 | p = 0.87 |
|  | Epoch x device | F_5.9,1381.8_ = 6.48 | p <0001 * |
|  | Epoch x group x device | F_38.1,1381.8_ = 0.84 | p = 0.75 |

Supplementary Table 3. Statistical results of sex, handedness, and device on the initial and final angles. Statistically significant results are shown with an asterisk.

1. Moreno-Briseño, P., Díaz, R., Campos-Romo, A. & Fernandez-Ruiz, J. Sex-related differences in motor learning and performance. *Behav Brain Funct* **6**, 74 (2010).

2. Dorfberger, S., Adi-Japha, E. & Karni, A. Sex differences in motor performance and motor learning in children and adolescents: An increasing male advantage in motor learning and consolidation phase gains. *Behavioural Brain Research* **198**, 165–171 (2009).

3. Ruitenberg, M. F. L., Koppelmans, V., Seidler, R. D. & Schomaker, J. Developmental and age differences in visuomotor adaptation across the lifespan. *Psychological Research* **87**, 1710–1717 (2023).

4. Taylor, J. A., Krakauer, J. W. & Ivry, R. B. Explicit and Implicit Contributions to Learning in a Sensorimotor Adaptation Task. *J Neurosci* **34**, 3023–3032 (2014).

5. Benson, B. L., Anguera, J. A. & Seidler, R. D. A spatial explicit strategy reduces error but interferes with sensorimotor adaptation. *J Neurophysiol* **105**, 2843–2851 (2011).

6. Malone, L. A., Hill, N. M., Tripp, H., Wolpert, D. M. & Bastian, A. J. A novel video game for remote studies of motor adaptation in children. *Physiological Reports* **11**, e15764 (2023).
